# Gradient Flow Dynamics for Cell Membranes in the Canham–Helfrich Model

**DOI:** 10.1007/s00205-026-02235-y

**Published:** 2026-08-24

**Authors:** Fabian Rupp, Christian Scharrer, Manuel Schlierf

**Affiliations:** 1 Faculty of Mathematics, University of Vienna, Oskar-Morgenstern-Platz 1, 1090 Vienna, Austria; 2 Institute for Applied Mathematics, University of Bonn, Endenicher Allee 60, 53115 Bonn, Germany; 3 Salzburg University, Hellbrunner Str. 34, 5020 Salzburg, Austria

## Abstract

The most energetically efficient way that a deformed red blood cell regains equilibrium is mathematically described by the gradient flow of the Canham–Helfrich functional, including a spontaneous curvature and the conservation of surface area and enclosed volume. Using a recently discovered multiplicity inequality, we prove the global existence and convergence of smooth solutions for spheres and axisymmetric tori, provided that the initial energy lies below explicit thresholds.

## Introduction

We consider the bending energy of an immersed surface $f:\Sigma \rightarrow {\mathbb {R}}^3$ defined by

1.1 $$\begin{aligned} {\mathcal {W}}(f):= \frac{1}{4}\int _{\Sigma }H^2\,\mathrm d\mu , \end{aligned}$$

where $H := \kappa _1+\kappa _2$ is the mean curvature, and μ is the Riemannian measure on Σ induced by the pull back metric $g_f$. This energy functional is not only *scale-invariant,* but even invariant under all conformal transformations of the ambient space, as already observed by Blaschke and Thomsen [12, 57]. The energy ${\mathcal {W}}$ became more popular after the work of Willmore [55] who also showed that among all immersions of closed surfaces, the round spheres are the absolute minimizers with energy 4π [56]. An essential tool in examining the Willmore energy is an inequality of Li and Yau [34] relating the energy with the multiplicity at any point. That is, if $p\in {\mathbb {R}}^3$ with $f^{-1}(p)=\{x_1,\ldots ,x_k\}$, $x_i\ne x_j$ for i≠j, then

1.2 $$\begin{aligned} 4\pi k \leqq {\mathcal {W}}(f). \end{aligned}$$

In particular, the condition ${\mathcal {W}}(f)<8\pi$ rules out points of higher multiplicity, implying that *f* is an embedding. Using the conformal invariance, Bryant [11] proved that *Willmore spheres*, that is, spheres critical for (1.1), always attain equality in (1.2) and arise from the stereographic projection of minimal surfaces in ${\mathbb {S}}^3$. In particular, he proved that the only Willmore spheres with energy strictly less than 8π are the round spheres. This fact should become a key ingredient in the works of Kuwert and Schätzle on the *Willmore flow*, the $L^2$-gradient flow of the Willmore functional [29–31]. Unless additional symmetry is assumed [19, 23], the only generic long time existence result is for topological spheres due to the lack of an analogue of Bryant’s classification result.

### The Stationary Canham–Helfrich Model

One of the most prominent applications of the Willmore energy is the connection to biological membranes. In the 70 s, Helfrich [21] derived the curvature elastic energy per unit area of a lipid bilayer

1.3 $$\begin{aligned} \frac{1}{2}k_c(H-c_0)^2 + {\bar{k}}_cK, \end{aligned}$$

where $k_c,{\bar{k}}_c$ are curvature elastic moduli, see also the previous work of Canham [13]. The so-called spontaneous curvature $c_0\in {\mathbb {R}}$ is a parameter that allows the model to be adjusted with respect to both, the type of membrane as well as its two aqueous solutions. For the vital example of red blood cells, this parameter was found to be negative and large in modulus [17]. The membrane itself is modeled as an immersed surface $f:\Sigma \rightarrow {\mathbb {R}}^3$ whose total stored energy is obtained by integrating (1.3) over Σ, where the Gauss–Bonnet theorem implies that the integral of ${\bar{k}}_cK$ is a topological constant. Introducing $c_0\ne 0$ reduces the invariance group of the functional compared to the Willmore functional (1.1), see [18], so that previous methods based on conformal invariance [11, 34] are no longer available. Moreover, the energy is invariant only under *positive* (that is, orientation-preserving) reparametrizations. Additional complexity arises from the inextensibility of the membrane and the osmotic pressure balance between the cell and its surroundings that are mathematically modeled by prescribing the area and volume. Given $c_0\in {\mathbb {R}}$, $g\in {\mathbb {N}}_0$, $A_0>0$, and $0<V_0 < A_0^{3/2}/\sqrt{36\pi }$, we thus arrive at the minimization problem

HP $$\begin{aligned} \begin{aligned}&\textrm{Minimize}\qquad {\mathcal {H}}_{c_0}(f):=\frac{1}{4}\int _\Sigma (H-c_0)^2\,\mathrm d\mu , \\&\text {among smooth immersions }f:\Sigma \rightarrow {\mathbb {R}}^3 \text { with } \operatorname {genus}\Sigma =g,\ {\mathcal {A}}(f)=A_0,\ \\&\quad \text { and }{\mathcal {V}}(f)=V_0, \end{aligned} \end{aligned}$$

where ${\mathcal {A}}$ and ${\mathcal {V}}$ denote the area and volume.

The existence of solutions for $c_0=0$, g=0 was proven by Schygulla [49]. For nonzero spontaneous curvature, Mondino and the second author [37] showed that solutions do exist in general, but are not always given by smooth submanifolds. Indeed, a bubbling phenomenon may occur, cf. [43, Example 1.2]. For $c_0=0$ and $g\in {\mathbb {N}}_0$ arbitrary, existence of smooth minimizers has been proven recently, see [26, 27, 39, 50], whereas existence in weak settings (without prescribing the topology) has been obtained in [10, 25]. An explicit general criterion on the data $A_0,V_0,c_0$ that guarantees smoothly embedded spherical minimizers has been obtained by the first two authors [43] by generalizing the inequality of Li and Yau (1.2) for the Helfrich functional

1.4 $$\begin{aligned} 4k\pi \leqq {\mathcal {H}}_{c_0}(f) - 2c_0\int _\Sigma \frac{\langle f - p,\nu \rangle }{|f-p|^2}\,\mathrm d\mu , \end{aligned}$$

where ν is the unit normal induced by the orientation of Σ, cf. Section 2.1. If *f* is an Aleksandrov immersion (see [43, Definition 1.4]) or a volume varifold (see [43, Hypothesis 4.5], [51, Definition 3.1]), the last integral can be controlled in terms of the energy ${\mathcal {H}}_{c_0}$ and the data $A_0,V_0,c_0$, by means of the diameter bounds in [43, 51]. Consequently, smooth solutions for the Helfrich problem (HP) do exist, provided the minimal energy satisfies that

1.5 $$\begin{aligned} \inf _{f}{\mathcal {H}}_{c_0}(f) < \Omega (A_0,V_0,c_0), \end{aligned}$$

where $\Omega (A_0,V_0,c_0)$ is defined as in Definition 2.6 below.

### Dynamic Approach and Main Results

Capillaries, the smallest human blood vessels, can be so thin that red blood cells have to be deformed in order to fit through. Classical experiments show how red blood cells deform when sucked into a micropipette [42]. After being released, the cells regain their original shape within seconds. In a viscous regime, the continuous deformation towards an energetically more favorable state can be schematically described by the $L^2$-gradient flow of the energy. Given an initial immersion $f_0:{\mathbb {S}}^2\rightarrow {\mathbb {R}}^3$, a family of immersions $f:[0,T)\times {\mathbb {S}}^2\rightarrow {\mathbb {R}}^3$ with $f(0,\cdot )=f_0$ evolves according to the $L^2$-gradient flow of the Helfrich functional if

1.6 $$\begin{aligned} \partial _t f = - 2\Pi (f) \nabla {\mathcal {H}}_{c_0}(f), \end{aligned}$$

where Π(f) is the $L^2(\textrm{d}\mu _f)$-orthogonal projection corresponding to the tangent space of the manifold of immersions with prescribed area and volume. This projection makes (1.6) a quasilinear nonlocal parabolic system, which is degenerate due to its geometric invariance. Moreover, being of fourth order, maximum principles are not available. Whenever the mean curvature is not constant on Σ, that is, the *CMC-deficit*

1.7 $$\begin{aligned} \overline{{\mathcal {H}}}(f) := \frac{1}{4}\int _\Sigma (H-{\overline{H}})^2\textrm{d}\mu \end{aligned}$$

is strictly positive, Equation (1.6) can be equivalently written as

1.8 $$\begin{aligned} \partial _t f = - \big ( 2\nabla {\mathcal {H}}_{c_0}(f) + \lambda _1(f)\nabla {\mathcal {A}}(f) + \lambda _2(f)\nabla {\mathcal {V}}(f)\big ), \end{aligned}$$

where the two Lagrange-multipliers $\lambda _1,\lambda _2$ are uniquely determined by the condition that area and volume are preserved along the flow, see (2.8) and (2.9) in Section 2.3. Any smooth solution $f:[0,T)\times \Sigma \rightarrow {\mathbb {R}}^3$ of (1.8) with constant area $A_0\equiv {\mathcal {A}}(f)$ and constant volume $V_0\equiv {\mathcal {V}}(f)$ will be referred to as an $(A_0,V_0,c_0)$*-Helfrich flow*. The short time existence and uniqueness has been proven by Nagasawa and Yi [41]. In this paper, we prove global existence and convergence if the initial energy is below an explicit threshold determined by the parameters.

#### Theorem 1.1

Consider $$A_0,V_0>0$$ with

1.9 $$\begin{aligned} \sigma := 36\pi \frac{V_0^2}{A_0^3} \in (0,1), \end{aligned}$$

$c_0\in {\mathbb {R}}$, and let $f_0:{\mathbb {S}}^2\rightarrow {\mathbb {R}}^3$ be an embedding with ${\mathcal {A}}(f_0)=A_0$ and ${\mathcal {V}}(f_0)=V_0$, satisfying

1.10 $$\begin{aligned} \sqrt{{\mathcal {H}}_{c_0}(f_0)} \leqq \min \Bigl \{\sqrt{\Omega (A_0,V_0,c_0)}, \sqrt{\frac{4\pi }{\sigma }} - \frac{c_0}{2}\sqrt{A_0}\Bigr \}. \end{aligned}$$

Then, the $(A_0,V_0,c_0)$-Helfrich flow with initial datum $f_0$ exists globally, is embedded for all t≧0, and converges after positive reparametrization smoothly to an embedded *constrained* ${\mathcal {H}}_{c_0}$*-Helfrich sphere* $f_\infty :{\mathbb {S}}^2\rightarrow {\mathbb {R}}^3$ as $t\rightarrow \infty$, that is, to a solution of

1.11 $$\begin{aligned}&\Delta H + |A^0|^2H + c_0(\frac{1}{2}H^2-|A^0|^2) - (\lambda _1+\frac{1}{2}c_0^2) H - \lambda _2 = 0 \quad \nonumber \\&\quad \text { for some }\lambda _1,\lambda _2\in {\mathbb {R}}. \end{aligned}$$

The assumption of spherical topology in Theorem 1.1 is necessary in view of the absence of a classification result as in [11] for higher genus Willmore surfaces. However, if the initial surface is an axisymmetric torus, we may use the strategy from [19] to conclude the following:

#### Theorem 1.2

Let $A_0,V_0>0$ satisfy (1.9), $c_0\in {\mathbb {R}}$, and $f_0:{\mathbb {T}}^2\rightarrow {\mathbb {R}}^3$ be an axisymmetric embedded torus with ${\mathcal {A}}(f_0)=A_0$ and ${\mathcal {V}}(f_0)=V_0$, satisfying (1.10). Then, the $(A_0,V_0,c_0)$-Helfrich flow with initial datum $f_0$ exists globally, is embedded for all t≧0, and converges after positive reparametrization smoothly to an embedded axisymmetric constrained ${\mathcal {H}}_{c_0}$-Helfrich torus $f_\infty :{\mathbb {T}}^2\rightarrow {\mathbb {R}}^3$ as $t\rightarrow \infty$.

Whenever the parameters $A_0,V_0,c_0$ allow for an admissible initial datum for Theorems 1.1 and 1.2, we can conclude existence of critical points. Since

$$\begin{aligned} \lim _{c_0\rightarrow 0}{\mathcal {H}}_{c_0} = {\mathcal {W}},\qquad \lim _{c_0\rightarrow 0} \Omega (A_0,V_0,c_0) = 8\pi \end{aligned}$$

(see Lemma 2.1 for a quantitative upper bound of the first limit) we have by [48, Theorem 7.1] that for all $A_0,V_0>0$ satisfying (1.9), there exists $\varepsilon =\varepsilon (A_0,V_0)>0$ such that Condition (1.10) admits axisymmetric spherical initial embeddings $f_0$ whenever $|c_0|<\varepsilon$. In addition, these $f_0$ are symmetric with respect to a plane orthogonal to the axis of rotation.

On the other hand, since, for $c_0<0$,

$$\begin{aligned} \lim _{A_0\rightarrow 0}{\mathcal {H}}_{c_0} = {\mathcal {W}},\qquad \lim _{r\rightarrow 0+}\Omega (r^2A_0,r^3V_0,c_0) = 8\pi , \end{aligned}$$

(see Definition 2.6), one may use suitably scaled axisymmetric embedded spheres and tori constructed in [50, Theorem 1.2. and 1.1] to see that for all $c_0<0$ and for all $\sigma \in (0,1/2)$ there exist ${\bar{A}}_0,{\bar{V}}_0>0$ with $36\pi {\bar{V}}_0^2/{\bar{A}}_0^3 = \sigma$ such that for all $0<A_0<{\bar{A}}_0$, $0<V_0<{\bar{V}}_0$ with $36\pi V_0^2/A_0^3 = \sigma$, Condition (1.10) admits axisymmetric surfaces $f_0$. By uniqueness, the evolution equation (1.8) preserves axial (and reflection) symmetry, and thus we obtain

#### Corollary 1.3

Let $\Sigma \in \{{\mathbb {S}}^2,{\mathbb {T}}^2\}$ and suppose that $A_0,V_0>0$ satisfy (1.9). There exists $\varepsilon ^\Sigma (A_0,V_0)>0$ and ${\bar{A}}_0^\Sigma ,{\bar{V}}_0^\Sigma >0$ such that if $c_0\in {\mathbb {R}}$ and

$$\begin{aligned} |c_0|<\varepsilon ^\Sigma (A_0,V_0) \qquad \text { or } \qquad c_0<0,\; A_0<{\bar{A}}_0^\Sigma , \; V_0<{\bar{V}}_0^\Sigma , \; 36\pi V_0^2/A_0^3< 1/2, \end{aligned}$$

then there exists an axisymmetric constrained ${\mathcal {H}}_{c_0}$-Helfrich immersion $f_\infty :\Sigma \rightarrow {\mathbb {R}}^3$, which is symmetric with respect to a plane orthogonal to the axis of revolution, and satisfies ${\mathcal {A}}(f_\infty )=A_0$ and ${\mathcal {V}}(f_\infty )=V_0$.

Among axisymmetric surfaces of genus $g\in \{0,1\}$, existence of minimizers for (HP) has been shown in [16]. However, these minimizers do not need to be smooth, and thus our findings provide the first axisymmetric spheres and tori solving (1.11) for $c_0\ne 0$. In the case $c_0=0$, it follows from [35] that the minimizers of [16] are smooth.

### Relation to Previous Works

For general $c_0\in {\mathbb {R}}$, global existence of an energy-decreasing evolution for ${\mathcal {H}}_{c_0}$ that preserves both area and volume has recently been proven in [7]. Their approach is based on the theory of varifolds, a weak notion of surfaces, and De Giorgi’s *Generalized Minimizing Movements.* While allowing for very general and possible singular shapes, the physical interpretation of these varifold solutions is limited, since it is still unknown whether solutions that are constant in time or their limits as $t\rightarrow \infty$ are equilibria of the system. Instead, Theorems 1.1 and 1.2 provide a dynamic version of the Canham–Helfrich model (HP) that yields classical smooth solutions, converging to equilibria.

In contrast to the previous work [32] on local stability of minimizers under the flow, in Theorems 1.1 and 1.2 we do not assume the initial datum to be close, in parametrization, to a minimizer. Instead, (1.10) gives an explicit threshold, depending on the parameters, below which we have global existence and convergence. This is a common assumption for Willmore-type gradient flows, see [19, 22, 23, 31, 47, 48], which is also necessary, in general, by Blatt’s counterexample [8]. In [53], the third author considers the nonprojected analog of (1.6) where area and volume are not preserved along the evolution. While a positive result is obtained for $c_0>0$, in contrast to Theorem 1.1, in the physical case $c_0<0$ [17] the evolution of the nonprojected flow can shrink to a “round point” in finite time, see also [9, 40] for the case $c_0=0$. Thus, fixing area and volume along the flow prevents such undesirable finite-time singularities and eventually produces constrained critical points of ${\mathcal {H}}_{c_0}$ as a limit.

In literature, there are various computational approaches to the area- and volume-preserving Helfrich flow [3–6]. For instance, numerical simulations of $(A_0,V_0,0)$-Helfrich flows can be found in [3, Figures 14–17] and [6, Figure 5], where bi-concave surfaces are numerically obtained as limiting shapes of the flow already for $c_0=0$.

Sharpness of the energy threshold (1.10) is still an open problem. For the Willmore flow, the existence of singularities was first suggested by numerical experiments in [36] and only analytically verified years later in [8], using the classification of axisymmetric Willmore surfaces in [33]. Asymptotically, the initial data for the Willmore flow considered in the singular examples in [8, 36] approach the varifold $V_{\textrm{lim}}$ made up of two round spheres of radius 1 touching in a single point, up to scaling. One finds $A_0={\mathcal {A}}(V_{\textrm{lim}})=8\pi$, $V_0={\mathcal {V}}(V_{\textrm{lim}})=\frac{8}{3}\pi$ and $\sigma =\frac{1}{2}$, using (1.9). Thus, for $c_0\in (-\infty ,2]$,

$$\begin{aligned} \sqrt{{\mathcal {H}}_{c_0}(V_{\textrm{lim}})} = \sqrt{8\pi }-\frac{c_0}{2}\sqrt{8\pi } \geqq \min \Bigl \{\sqrt{\Omega (A_0,V_0,c_0)}, \sqrt{\frac{4\pi }{\sigma }} - \frac{c_0}{2}\sqrt{A_0}\Bigr \}, \end{aligned}$$

with equality for $c_0\in [0,2]$ so that our energy threshold (1.10) is attained in this case. Whether the initial data considered in [8, 36] actually result in singular $(A_0,V_0,c_0)$-Helfrich flows is still an open problem. However, for the nonprojected analog of (1.6) analytically treated in [53], there is numerical evidence in this direction. Indeed, numerical experiments in [4, Section 5.3, Figure 9] suggest that, for $c_0=2$, starting in a suitable spherical “tube”, one has convergence (in a weak sense) of the nonprojected analog of (1.6) to $V_{\textrm{lim}}$.

### New Challenges and Strategy of the Proofs

In the singularity analysis of (1.8), there are several new difficulties to overcome compared to previous works on (constrained) *Willmore* flows, cf. in particular [48]. First, in general, the classical Li–Yau inequality (1.2) cannot be used to conclude embeddedness from smallness of the Helfrich energy, see [43, Example 1.2]. Fortunately, the perfectly suited Li–Yau-type inequality (1.4) was proven in [43] for exactly this purpose. Since the limit of the flow in Theorem 1.1 is in particular a smooth constrained critical point for (HP), the parameters $A_0,V_0,c_0$ should be in the same range as in the corresponding stationary problem (1.5), hence (1.10) comprises the assumption

$$\begin{aligned} {\mathcal {H}}_{c_0}(f_0) \leqq \Omega (A_0,V_0,c_0). \end{aligned}$$

Second, the degeneracy of the constraints in (1.8) needs to be controlled by proving a uniform lower bound on (1.7). This corresponds to a *quantitative Aleksandrov inequality* (see [15, 24]) which cannot be simply achieved by the triangle inequality in contrast to [48, Lemma 4.1]. Third, to obtain a *localized curvature concentration-based criterion* à la Kuwert–Schätzle [30], appropriate *a priori estimates* that reduce the order of the scaling-critical nonlocal Lagrange multipliers in (1.8) are needed. For this purpose, the condition (1.10) ensures that

$$\begin{aligned} \sqrt{{\mathcal {H}}_{c_0}(f_0)}\leqq \sqrt{\frac{4\pi }{\sigma }} - \frac{c_0}{2}\sqrt{A_0}, \end{aligned}$$

simplifying to ${\mathcal {W}}(f_0)\leqq 4\pi /\sigma$ if $c_0=0$ which is in accordance with [48]. Fourth, we would like to use the removability result in [31] to apply [11] in the analysis of noncompact parabolic blow-up limits. However, this requires control over quantities appearing in the monotonicity formula for the Willmore energy [31, 54] that can generically not be controlled by the Helfrich energy. Last, it is crucial to preserve orientation during all reparametrization processes to ensure the invariance of both the energy and the flow.

To prove Theorem 1.1, we first follow the methods of Kuwert and Schätzle [29, 30] and study a localized version of the energy in which the Lagrange multipliers appear, see Section 3. Exploiting the scaling behavior of the involved geometric quantities, we obtain a linear system for $\lambda _1,\lambda _2$, see Lemma 4.1. Under the assumption (1.10), this allows us to reduce the order of the Lagrange multipliers to obtain a concentration-based lifespan bound similar to [30] in Proposition 5.3. We then follow [29, 31] to construct a blow-up, which we can identify as a (possibly noncompact) and nonplanar Helfrich immersion. Crucially, we find that whenever this blow-up limit is noncompact, it has to be a Willmore immersion, see Proposition 5.6, whereas if it is compact, we may argue as in [14] to conclude convergence employing an appropriate Łojasiewicz inequality [45] in Section 6. In Section 7, we exclude the possibility of noncompact blowups under the assumption (1.10). This follows from a precise examination of the scaling behavior of the monotonicity formula for the Helfrich functional from [43], which enables us to apply the removability result from [31] and then Bryant’s classification [11]. Lastly, in Section 8, we prove Theorem 1.2. To that end, we use the relation with elastic curves in the hyperbolic plane as in [19] and the observation that unbounded hyperbolic length necessarily produces a self-intersection on the axis of revolution [52] which is excluded by the Li–Yau inequality (1.4)

## Geometric Preliminaries

### Basic Definitions

Throughout this article, Σ denotes a closed, oriented and connected surface. For an immersion $f:\Sigma \rightarrow {\mathbb {R}}^3$ of Σ, $g:=f^*\langle \cdot ,\cdot \rangle$ denotes the pull-back metric on Σ, that is, $g_{ij}:=\langle \partial _i f,\partial _j f\rangle$ in local coordinates, with induced measure μ. Further, $\nu :\Sigma \rightarrow {\mathbb {S}}^2$ is the unique smooth unit normal field induced by the orientation of Σ. In local coordinates with respect to the orientation of Σ, one has

2.1 $$\begin{aligned} \nu :=\frac{\partial _1 f\times \partial _2 f}{|\partial _1 f\times \partial _2 f|}. \end{aligned}$$

The (scalar) second fundamental form of *f* is the (2, 0)-tensor determined by $A_{ij}:=\langle \partial ^2_{ij}f,\nu \rangle$ in local coordinates. Its trace is the mean curvature $H:=g^{ij}A_{ij}$ where $(g^{ij}) := (g_{ij})^{-1}$. The trace-free second fundamental form is given by $A_{ij}^0:=A_{ij}-\frac{1}{2} H g_{ij}$. Its squared $L^2(\textrm{d}\mu )$-norm is denoted by ${\mathcal {W}}_0(f):=\int _{\Sigma }|A^0|^2\textrm{d}\mu$. Using these definitions, one finds the relation

2.2 $$\begin{aligned} |A|^2&=|A^0|^2+\frac{1}{2} H^2. \end{aligned}$$

For any $c_0\in {\mathbb {R}}$, define the Helfrich energy with spontaneous curvature $c_0$

$$\begin{aligned} {\mathcal {H}}_{c_0}(f):=\frac{1}{4}\int _{\Sigma }(H-c_0)^2\textrm{d}\mu , \end{aligned}$$

where in the special case $c_0=0$, ${\mathcal {W}}(f)={\mathcal {H}}_{0}(f)$ is also called Willmore energy. Note that, as a consequence of the Gauss-Bonnet theorem, using that the Gauss curvature *K* can be written as $K=\frac{1}{4}H^2 - \frac{1}{2}|A^0|^2$, one has

2.3 $$\begin{aligned} {\mathcal {W}}_0(f)= \int _{\Sigma }|A^0|^2\textrm{d}\mu = 2{\mathcal {W}}(f)-8\pi (1-\operatorname {genus}\Sigma ). \end{aligned}$$

One further defines

$$\begin{aligned} {\mathcal {A}}(f)=\mu (\Sigma ),\quad {\mathcal {V}}(f)=-\frac{1}{3}\int _{\Sigma }\langle f,\nu \rangle \textrm{d}\mu ,\quad {\mathcal {I}}(f)=36\pi \frac{{\mathcal {V}}(f)^2}{{\mathcal {A}}(f)^3} \end{aligned}$$

as the area, signed volume, and isoperimetric ratio of *f*, respectively. Throughout this article, the orientation of Σ is chosen such that ${\mathcal {V}}(f)\geqq 0$. In particular, if *f* is an embedding, ν is inward pointing so that round spheres of radius r>0 have constant mean curvature H≡2/r>0.

#### Lemma 2.1

Let $c_0\in {\mathbb {R}}$ and consider an immersion $f:\Sigma \rightarrow {\mathbb {R}}^3$. Then

2.4 $$\begin{aligned} {\mathcal {W}}(f)\leqq \Bigl (\sqrt{{\mathcal {H}}_{c_0}(f)}+\frac{1}{2}\sqrt{c_0^2{\mathcal {A}}(f)}\Bigr )^2. \end{aligned}$$

#### Proof

Without loss of generality, suppose $c_0\ne 0$. By a direct computation, one finds for any $\varepsilon \in (0,1)$

$$\begin{aligned} {\mathcal {W}}(f)&= {\mathcal {H}}_{c_0}(f) + \frac{1}{2} c_0\int _{\Sigma } H\textrm{d}\mu - \frac{1}{4}c_0^2{\mathcal {A}}(f) \leqq {\mathcal {H}}_{c_0}(f) \\&\quad + \sqrt{{\mathcal {W}}(f)}\sqrt{c_0^2{\mathcal {A}}(f)} - \frac{1}{4}c_0^2{\mathcal {A}}(f)\\&\leqq {\mathcal {H}}_{c_0}(f) + \varepsilon {\mathcal {W}}(f) + \frac{1}{4}\frac{1-\varepsilon }{\varepsilon } c_0^2{\mathcal {A}}(f), \end{aligned}$$

by Young’s inequality. This yields

$$\begin{aligned} {\mathcal {W}}(f) \leqq \frac{1}{1-\varepsilon } {\mathcal {H}}_{c_0}(f) + \frac{1}{4\varepsilon } c_0^2{\mathcal {A}}(f). \end{aligned}$$

Minimizing with respect to $\varepsilon \in (0,1)$ yields the claim. □

### Evolution Equations

First, recall the following well-known evolutions of the relevant geometric quantities.

#### Lemma 2.2

For a family of immersions $f:[0,T)\times \Sigma \rightarrow {\mathbb {R}}^3$ with normal speed $\partial _t f=:\xi \nu$, one has in the coordinates of a local orthonormal frame ${e_1,e_2}$

2.5 $$\begin{aligned} \partial _t \textrm{d}\mu&= -H\xi \textrm{d}\mu \end{aligned}$$

2.6 $$\begin{aligned} \partial _t H&= \Delta \xi + |A|^2\xi =\Delta \xi + |A^0|^2\xi + \frac{1}{2}H^2\xi \end{aligned}$$

As in [48, Proposition 2.4], these evolutions immediately yield

#### Proposition 2.3

Let $f:\Sigma \rightarrow {\mathbb {R}}^3$ be an immersion and $\varphi :\Sigma \rightarrow {\mathbb {R}}^3$. One has the following first variation identities:

$$\begin{aligned} {\mathcal {W}}_0'(f)(\varphi )&=\int _{\Sigma } \langle [\Delta H+|A^0|^2H]\nu ,\varphi \rangle \textrm{d}\mu =:\langle \nabla {\mathcal {W}}_0(f),\varphi \rangle _{L^2(\textrm{d}\mu )},\\ {\mathcal {A}}'(f)(\varphi )&=-\int _{\Sigma } \langle H\nu ,\varphi \rangle \textrm{d}\mu =:\langle \nabla {\mathcal {A}}(f),\varphi \rangle _{L^2(\textrm{d}\mu )},\\ {\mathcal {V}}'(f)(\varphi )&=-\int _{\Sigma } \langle \nu ,\varphi \rangle \textrm{d}\mu =:\langle \nabla {\mathcal {V}}(f),\varphi \rangle _{L^2(\textrm{d}\mu )}, \end{aligned}$$

and finally, ${\mathcal {H}}_{c_0}'(f)(\varphi ) = \langle \nabla {\mathcal {H}}_{c_0}(f),\varphi \rangle _{L^2(\textrm{d}\mu )}$ with

$$\begin{aligned} 2\nabla {\mathcal {H}}_{c_0}(f)&=\nabla {\mathcal {W}}_0(f)-c_0|A^0|^2\nu + \frac{1}{2}c_0H(H-c_0)\nu \\&= [\Delta H + |A^0|^2(H-c_0) + \frac{1}{2} c_0 H(H-c_0)]\nu . \end{aligned}$$

### The Area and Volume Preserving Helfrich Flow

Given $A_0,V_0>0$ with (1.9) and $c_0\in {\mathbb {R}}$, a family of immersions $f\in C^\infty ([0,T)\times \Sigma ;{\mathbb {R}}^3)$ satisfying $A_0={\mathcal {A}}(f(0))$, $V_0={\mathcal {V}}(f(0))$ and

2.7 $$\begin{aligned} \partial _t f&= -\Bigl [ \Delta H + |A^0|^2H + c_0(\frac{1}{2}H^2-|A^0|^2) - (\lambda _1+\frac{1}{2}c_0^2) H - \lambda _2 \Bigr ]\nu \nonumber \\&= -2 \nabla {\mathcal {H}}_{c_0}(f) + \lambda _1(f) H\nu + \lambda _2(f) \nu \end{aligned}$$

where

2.8 $$\begin{aligned} \lambda _1:= \lambda _1(f)=2\frac{{\mathcal {A}}(f)\int _{\Sigma }\langle \nabla {\mathcal {H}}_{c_0}(f),H\nu \rangle \textrm{d}\mu -\int _{\Sigma }H\textrm{d}\mu \int _{\Sigma }\langle \nabla {\mathcal {H}}_{c_0}(f),\nu \rangle \textrm{d}\mu }{4{\mathcal {W}}(f){\mathcal {A}}(f)-(\int _{\Sigma }H\textrm{d}\mu )^2} \end{aligned}$$

and

2.9 $$\begin{aligned} \lambda _2:=\lambda _2(f)=2\frac{-\int _{\Sigma }H\textrm{d}\mu \int _{\Sigma }\langle \nabla {\mathcal {H}}_{c_0}(f),H\nu \rangle \textrm{d}\mu +4{\mathcal {W}}(f)\int _{\Sigma }\langle \nabla {\mathcal {H}}_{c_0}(f),\nu \rangle \textrm{d}\mu }{4{\mathcal {W}}(f){\mathcal {A}}(f)-(\int _{\Sigma }H\textrm{d}\mu )^2} \end{aligned}$$

is referred to as an $(A_0,V_0,c_0)$-Helfrich flow starting in $f_0:=f(0)$.

#### Remark 2.4

By direct computation, if $f:[0,T)\times \Sigma \rightarrow {\mathbb {R}}^3$ is an $(A_0,V_0,c_0)$-Helfrich flow, then ${\mathcal {A}}(f(t))=A_0$ and ${\mathcal {V}}(f(t))=V_0$ for all t∈[0,T). Moreover,

2.10 $$\begin{aligned} 2\frac{\textrm{d}}{\textrm{d}t}{\mathcal {H}}_{c_0}(f(t)) = 2 \int _{\Sigma } \langle \nabla {\mathcal {H}}_{c_0}(f),\partial _tf\rangle \textrm{d}\mu =-\int _{\Sigma } |\partial _tf|^2\textrm{d}\mu \leqq 0. \end{aligned}$$

Particularly, $t\mapsto {\mathcal {H}}_{c_0}(f(t))$ is nonincreasing and thus $\lim _{t\nearrow T}{\mathcal {H}}_{c_0}(f(t))$ exists. Moreover, for a *positive*, that is, orientation preserving diffeomorphism $\phi :\Sigma \rightarrow \Sigma$, ${\tilde{f}}:[0,T)\times \Sigma \rightarrow {\mathbb {R}}^3$ with ${\tilde{f}}(t,x):=f(t,\phi (x))$ again is an $(A_0,V_0,c_0)$-Helfrich flow.

#### Remark 2.5

Writing , we observe that the denominator in (2.8) and (2.9) satisfies

$$\begin{aligned} 4{\mathcal {W}}(f)& {\mathcal {A}}(f)-\Bigl (\int _{\Sigma }H\textrm{d}\mu \Bigr )^2 = \frac{1}{2} \int _{\Sigma }\int _{\Sigma } \bigl (H(x)-H(y)\bigr )^2\textrm{d}\mu (x)\textrm{d}\mu (y) \\&= {\mathcal {A}}(f)\Big (\int _{\Sigma }H^2\textrm{d}\mu -{\mathcal {A}}(f){\overline{H}}^2\Big ) = {\mathcal {A}}(f)\int _{\Sigma }(H-{\overline{H}})^2\textrm{d}\mu , \end{aligned}$$

so that, with $\overline{{\mathcal {H}}}(f)$ as in (1.7),

2.11 $$\begin{aligned} 4{\mathcal {W}}(f){\mathcal {A}}(f)-\Bigl (\int _{\Sigma }H\textrm{d}\mu \Bigr )^2 = 4{\mathcal {A}}(f){\overline{{\mathcal {H}}}}(f). \end{aligned}$$

By the famous Aleksandrov theorem [2], this vanishes for embedded surfaces only in case of the round sphere. This however is impossible if $36\pi {\mathcal {V}}(f)^2<{\mathcal {A}}(f)^3$ by the isoperimetric theorem.

We now define the constant Ω that appears in Theorem 1.1.

#### Definition 2.6

For all $c_0\in {\mathbb {R}}$ and $A_0,V_0>0$ define

$$\begin{aligned} \Omega (A_0,V_0,c_0):= {\left\{ \begin{array}{ll} 4\pi +\sqrt{(4\pi )^2+\frac{|c_0|V_0}{2C^2A_0}}& \hbox { if}\ c_0<0 \\ \max \{(\sqrt{8\pi }-\frac{1}{2}\sqrt{c_0^2A_0})_+^2,8\pi -6c_0(4\pi ^2V_0)^{\frac{1}{3}}\}& \text {if }c_0\ge 0 \end{array}\right. } \end{aligned}$$

where *C* is the best constant in [51, Theorem 1.2], and $(x)_+:=\max \{0,x\}$ denotes the positive part of $x\in {\mathbb {R}}$.

The constant $\Omega (A_0,V_0,c_0)$ is chosen to control the right hand side in the Li–Yau-type inequality (1.4).

#### Lemma 2.7

Let δ>0, $f:\Sigma \rightarrow {\mathbb {R}}^3$ be an embedding, and $A_0:={\mathcal {A}}(f)$, $V_0:={\mathcal {V}}(f)$ satisfy (1.9). Suppose that ${\mathcal {H}}_{c_0}(f)\le \Omega (A_0,V_0,c_0)-\delta$. If $c_0\geqq 0$ and $\Omega (A_0,V_0,c_0)= (\sqrt{8\pi }-\frac{1}{2}\sqrt{c_0^2A_0})_+^2$, then

2.12 $$\begin{aligned} \frac{1}{4\pi }{\mathcal {W}}(f) \leqq 2-\frac{\delta }{4\pi }. \end{aligned}$$

Otherwise

2.13 $$\begin{aligned} \frac{1}{4\pi } {\mathcal {H}}_{c_0}(f) - \frac{c_0}{2\pi }\int _\Sigma \frac{\langle f-x_0,\nu \rangle }{|f-x_0|^2}\textrm{d}\mu \le 2-\frac{\delta }{4\pi }. \end{aligned}$$

#### Proof

Suppose $c_0\geqq 0$ and $\Omega (A_0,V_0,c_0)= (\sqrt{8\pi }-\frac{1}{2}\sqrt{c_0^2A_0})_+^2$. Noting that $\Omega (A_0,V_0,c_0)>{\mathcal {H}}_{c_0}(f)\geqq 0$, Lemma 2.1 implies (2.12).

If $c_0\ge 0$ and $\Omega (A_0,V_0,c_0)= 8\pi - 6 c_0 (4\pi ^2 V_0)^{\frac{1}{3}}$, then [43, Remark 6.11(ii)] implies (2.13).

Lastly, for $c_0\leqq 0$, we use the divergence theorem (cf. [43, (6.18)]) combined with the diameter estimate [51, Theorem 1.2], to infer

$$\begin{aligned} -\int _\Sigma \frac{\langle f-x_0,\nu \rangle }{|f-x_0|^2}\textrm{d}\mu \ge \frac{V_0}{4C^2 A_0{\mathcal {H}}_{c_0}(f)}. \end{aligned}$$

Hence, since $c_0\leqq 0$, we estimate

$$\begin{aligned} \frac{1}{4\pi } {\mathcal {H}}_{c_0}(f) - \frac{c_0}{2\pi }\int _\Sigma \frac{\langle f-x_0,\nu \rangle }{|f-x_0|^2}\textrm{d}\mu \leqq \frac{\Omega -\delta }{4\pi } + \frac{c_0}{2\pi }\frac{V_0}{4C^2 A_0 \Omega }. \end{aligned}$$

A straightforward computation shows that with $\Omega =\Omega (A_0,V_0,c_0)$ as in Definition 2.6 for $c_0<0$, we have

$$\begin{aligned} \frac{\Omega }{4\pi } + \frac{c_0}{2\pi }\frac{V_0}{4C^2 A_0 \Omega } = 2, \end{aligned}$$

and (2.13) follows. □

#### Proposition 2.8

Let r>0. For $\Omega (A_0,V_0,c_0)$ as in Definition 2.6, we have

$$\begin{aligned} \Omega \Big (\frac{A_0}{r^2},\frac{V_0}{r^3},rc_0\Big ) = \Omega (A_0,V_0,c_0). \end{aligned}$$

Moreover, for any smoothly immersed oriented surface $f:\Sigma \rightarrow {\mathbb {R}}^3$, it holds that

2.14 $$\begin{aligned} {\mathcal {H}}_{rc_0}(r^{-1}f)={\mathcal {H}}_{c_0}(f). \end{aligned}$$

#### Proposition 2.9

Let $f_0:\Sigma \rightarrow {\mathbb {R}}^3$ be a smooth embedding with $A_0={\mathcal {A}}(f_0)$ and $V_0={\mathcal {V}}(f_0)$ satisfying

$$\begin{aligned} {\mathcal {H}}_{c_0}(f_0)<\Omega (A_0,V_0,c_0) \end{aligned}$$

for some $c_0\in {\mathbb {R}}$. Let $f:[0,T)\times \Sigma \rightarrow {\mathbb {R}}^3$ be an $(A_0,V_0,c_0)$-Helfrich flow starting in $f_0$. Then $f(t,\cdot ):\Sigma \rightarrow {\mathbb {R}}^3$ is an embedding for all t∈[0,T).

#### Proof

Let

$$\begin{aligned} I:=\{t\in [0,T)\mid f(t,\cdot )\text { is an embedding}\}. \end{aligned}$$

A simple proof by contradiction using [1, Lemma 6.19] shows that *I* is open. Moreover, $I\ne \emptyset$ since 0∈I. Thus, it is enough to show that *I* is closed. To this end, let $t_0 \in (0,T)$ be in the closure of *I*. Let $V_t$ with t∈I be the oriented varifolds induced by f(t,·), cf. [43, Example 2.4]. By the Jordan–Brouwer separation theorem, all $V_t$ are volume varifolds in the sense of [51, Definition 3.1], cf. [43, Lemma 6.6]. Combining (2.2)(2.3) with Lemma 2.1 and Remark 2.4, we see that the second fundamental forms of $V_t$ remain bounded in $L^2$ uniformly in *t*. Thus, by [51, Theorem 7.6] there exists a varifold limit $V_{\infty }$ for a subsequence $t_k\rightarrow t_0$ which is again a volume varifold satisfying

$$\begin{aligned} {\mathcal {H}}_{c_0}(V_\infty )<\Omega (A_0,V_0,c_0). \end{aligned}$$

Since $f(t_k,\cdot )\rightarrow f(t_0,\cdot )$ in $C^1(\Sigma )$, we see that $V_\infty$ is the varifold induced by $f(t_0,\cdot )$. Now, [43, Corollary 4.11] implies that $f(t_0,\cdot )$ is an embedding. Consequently, $t_0\in I$ and *I* is closed. □

Using (2.11), we control the denominators in (2.8) and (2.9) with the following

#### Proposition 2.10

Let $c_0\in {\mathbb {R}}$, $A_0,V_0>0$ satisfy (1.9), and ε>0. Then, denoting the set of smooth embeddings $f:\Sigma \rightarrow {\mathbb {R}}^3$ with ${\mathcal {A}}(f)=A_0$ and ${\mathcal {V}}(f)=V_0$ by ${\mathcal {S}}_\Sigma (A_0,V_0)$, there holds

$$\begin{aligned}  &   {\bar{\eta }}_\varepsilon (A_0,V_0,c_0,\operatorname {genus}\Sigma )\\  &   \quad :=\inf \Bigl \{ {\overline{{\mathcal {H}}}}(f) \mid f\in {\mathcal {S}}_\Sigma (A_0,V_0),\,{\mathcal {H}}_{c_0}(f)\le \Omega (A_0,V_0,c_0)-\varepsilon \Bigr \}>0. \end{aligned}$$

#### Proof

For the sake of contradiction, assume the existence of a sequence $f_k\in {\mathcal {S}}_\Sigma (A_0,V_0)$ such that

$$\begin{aligned} \sup _{k\in {\mathbb {N}}}{\mathcal {H}}_{c_0}(f_k)\leqq \Omega (A_0,V_0,c_0) - \varepsilon \end{aligned}$$

and

$$\begin{aligned} \lim _{k\rightarrow \infty }{{\overline{{\mathcal {H}}}}}(f_k)=0. \end{aligned}$$

The total mean curvature is uniformly bounded:

$$\begin{aligned} A_0|{{\overline{H}}}_k| = \Big |\int _\Sigma H_k\textrm{d}\mu _k\Big |\leqq \sqrt{4A_0\Omega (A_0,V_0,c_0)} + |c_0|A_0. \end{aligned}$$

Thus, by passing to a subsequence, we achieve that the limit

$$\begin{aligned} \lim _{k\rightarrow \infty }{{\overline{H}}}_k=:h_0\in {\mathbb {R}}\end{aligned}$$

exists. Moreover,

$$\begin{aligned} {\mathcal {H}}_{h_0}(f_k)={{\overline{{\mathcal {H}}}}}(f_k) + \frac{1}{2}\big ({{\overline{H}}}_k-h_0\big )\int _{\Sigma }H_k\textrm{d}\mu _k - \frac{1}{4}\big ({{\overline{H}}}_k^2- h_0^2\big )A_0. \end{aligned}$$

Now, we may apply [51, Theorem 7.6] to deduce the existence of a volume varifold *V* in ${\mathbb {R}}^3$ with ${\mathcal {A}}(V)=A_0$, ${\mathcal {V}}(V)=V_0$, and

$$\begin{aligned} {\mathcal {H}}_{h_0}(V) = 0,\qquad {\mathcal {H}}_{c_0}(V) \leqq \Omega (A_0,V_0,c_0)-\varepsilon . \end{aligned}$$

By [43, Corollary 4.11] and [44, Theorem 5.8], the varifold *V* is induced by a $W^{2,2}$-conformal Lipschitz embedding $F:\Sigma _0\rightarrow {\mathbb {R}}^3$ of a Riemann surface $\Sigma _0$. Hence, the property ${\mathcal {H}}_{h_0}(F) = 0$ implies that in local conformal coordinates defined on the disk $D\subset {\mathbb {R}}^2$,

2.15 $$\begin{aligned} \Delta F = h_0\partial _1F\times \partial _2F, \end{aligned}$$

where Δ is the flat Laplacian in ${\mathbb {R}}^2$. The right hand side is in $W^{1,2}(D)$ since *F* is Lipschitz continuous. Bootstrapping Equation (2.15), it follows that *F* is smooth by standard elliptic regularity, cf. Theorems 5.6.6 and 6.3.2 in [20]. Thus, Aleksandrov’s theorem [2] implies that *F* parametrizes a round sphere, contradicting the assumption $36\pi V_0^2/A_0^3 < 1$. □

The following scaling behavior of the flow and especially the Lagrange multipliers $\lambda _1$ and $\lambda _2$ is immediate from their respective definitions (also cf. [48, Lemma 2.8]):

#### Lemma 2.11

Let $c_0\in {\mathbb {R}}$, consider $A_0,V_0>0$ satisfying (1.9) and let r>0. If $f:[0,T)\times \Sigma \rightarrow {\mathbb {R}}^3$ is an $(A_0,V_0,c_0)$-Helfrich flow, then its parabolic rescaling ${\widetilde{f}}:[0,T/r^4)\times \Sigma \rightarrow {\mathbb {R}}^3$ with ${\widetilde{f}}(t,p)=\frac{1}{r}f(r^4t,p)$ satisfies

(i) ${\widetilde{f}}$ is an $(\frac{1}{r^2}A_0,\frac{1}{r^3}V_0,rc_0)$-Helfrich flow.
(ii) $$\widetilde{\lambda _1}(t)=r^2\lambda _1(r^4t)$$ and $$\widetilde{\lambda _2}(t)=r^3\lambda _2(r^4t)$$.
(iii) $$\int _0^T(\lambda _1(t))^2\textrm{d}t =\int _{0}^{T/r^4}(\widetilde{\lambda _1}(t))^2\textrm{d}t$$ and $$\int _0^T|\lambda _2(t)|^{\frac{4}{3}}\textrm{d}t =\int _{0}^{T/r^4}|\widetilde{\lambda _2}(t)|^\frac{4}{3}\textrm{d}t$$.

## Localized Energy Estimates

This section is devoted to the fundamental estimates which enable a suitable blow-up construction in later sections. To this end, following [29, 47, 48, Section 3 respectively], one localizes the energy decay of $\int |A|^2\textrm{d}\mu$ to obtain a life-span theorem and suitable uniform bounds on derivatives of the curvature as long as an energy concentration is controlled.

Similarly as in [29, 47, 48], consider ${\widetilde{\gamma }}\in C_c^{\infty }({\mathbb {R}}^3)$ with $|{\widetilde{\gamma }}|\leqq 1$ and define $\gamma ={\widetilde{\gamma }}\circ f$. Fix some Λ>0 such that $\Vert D{\widetilde{\gamma }}\Vert _{\infty }\le \Lambda$ and $\Vert D^2{\widetilde{\gamma }}\Vert _{\infty }\le \Lambda ^2$. As on [47, p. 7], one estimates

3.1 $$\begin{aligned} |\nabla \gamma |\leqq \Lambda \quad \text {and}\quad |\nabla ^2\gamma |\leqq C(\Lambda ^2+|A|\Lambda ). \end{aligned}$$

In the following, $C\in (0,\infty )$ is a universal constant that may change from line to line:

### Lemma 3.1

Let $A_0,V_0>0$ satisfy (1.9), $c_0\in {\mathbb {R}}$ and $f:[0,T)\times \Sigma \rightarrow {\mathbb {R}}^3$ be an $(A_0,V_0,c_0)$-Helfrich flow. With ${\tilde{\gamma }}$, γ as in (3.1), we have

$$\begin{aligned} \frac{\textrm{d}}{\textrm{d}t}&\int _{\Sigma } |A|^2\gamma ^4\textrm{d}\mu + \frac{3}{2}\int _{\Sigma }|\nabla {\mathcal {W}}_0(f)|^2\gamma ^4\textrm{d}\mu \\&\le C(|\lambda _1|+c_0^2)\int _{\Sigma }\bigl (|\nabla ^2H||A|+|A|^4\bigr )\gamma ^4+\Lambda |A|^3\gamma ^3\textrm{d}\mu \\&\quad +C|\lambda _2|\int _{\Sigma }|A|^{3}\gamma ^4+\Lambda |A|^2\gamma ^{3}\textrm{d}\mu \\&\quad + C\Lambda ^4\int _{\{\gamma >0\}}|A|^2\textrm{d}\mu +C\Lambda ^2\int _{\Sigma }|A|^4\gamma ^2\textrm{d}\mu + C c_0^2\int _{\Sigma }|A|^4\gamma ^4\textrm{d}\mu . \end{aligned}$$

The proof of Lemma 3.1 can be found in Appendix A. The experienced reader can obtain it from [48, Lemma 3.2], formally replacing $3 \lambda /{\mathcal {A}}$ with $\lambda _1+c_0^2/2$ and $-2\lambda /{\mathcal {V}}$ with $\lambda _2$, in combination with [53, Lemma 4.2] for the additional terms linear in $c_0$.

### Proposition 3.2

There exist universal constants $\varepsilon _0,\delta _0,C\in (0,\infty )$ such that, for any *f* and γ as in Lemma 3.1, if

$$\begin{aligned} \int _{\{\gamma >0\}} |A|^2\textrm{d}\mu <\varepsilon _0\quad \text {at some time }t\in [0,T), \end{aligned}$$

then at time *t* one has

$$\begin{aligned} \frac{\textrm{d}}{\textrm{d}t}&\int _{\Sigma }|A|^2\gamma ^4\textrm{d}\mu + \delta _0 \int _{\Sigma } \bigl ( |\nabla ^2 A|^2+|A|^2|\nabla A|^2+|A|^6 \bigr )\gamma ^4\textrm{d}\mu \\&\leqq C\Lambda ^4\int _{\{\gamma >0\}}|A|^2\textrm{d}\mu + C(\lambda _1^2+|\lambda _2|^{\frac{4}{3}}+c_0^4)\int _{\Sigma }|A|^2\gamma ^4\textrm{d}\mu . \end{aligned}$$

### Proof

By [47, Proposition 3.2], see also [30, Proposition 2.6 and Lemma 4.2], at time *t*, the following interpolation inequality holds:

$$\begin{aligned} \int _{\Sigma } \bigl ( |\nabla ^2 A|^2\!+c|A|^2|\nabla A|^2\!+\!|A|^6 \bigr )\gamma ^4\textrm{d}\mu \!\leqq \! C\int _{\Sigma }|\nabla {\mathcal {W}}_0(f)|^2\gamma ^4\textrm{d}\mu \!+\!C\Lambda ^4\int _{\{\gamma \!>\!0\}}|A|^2\textrm{d}\mu . \end{aligned}$$

Using Lemma 3.1, there thus exists $\delta _0\in (0,\infty )$ with

$$\begin{aligned} \frac{\textrm{d}}{\textrm{d}t}&\int _{\Sigma } |A|^2\gamma ^4\textrm{d}\mu + 2\delta _0\int _{\Sigma } \bigl ( |\nabla ^2 A|^2+|A|^2|\nabla A|^2+|A|^6 \bigr )\gamma ^4\textrm{d}\mu \\&\leqq C(|\lambda _1|+c_0^2)\int _{\Sigma }\bigl (|\nabla ^2H||A|+|A|^4\bigr )\gamma ^4+\Lambda |A|^3\gamma ^3\textrm{d}\mu \\&\quad +C|\lambda _2|\int _{\Sigma }|A|^{3}\gamma ^4+\Lambda |A|^2\gamma ^{3}\textrm{d}\mu \\&\quad + C\Lambda ^4\int _{\{\gamma >0\}}|A|^2\textrm{d}\mu +C\Lambda ^2\int _{\Sigma }|A|^4\gamma ^2\textrm{d}\mu + C c_0^2\int _{\Sigma }|A|^4\gamma ^4\textrm{d}\mu . \end{aligned}$$

Proceeding as in [48, Proposition 3.3], one finds that

$$\begin{aligned} \frac{\textrm{d}}{\textrm{d}t}&\int _{\Sigma } |A|^2\gamma ^4\textrm{d}\mu + \frac{3}{2}\delta _0\int _{\Sigma } \bigl ( |\nabla ^2 A|^2+|A|^2|\nabla A|^2+|A|^6 \bigr )\gamma ^4\textrm{d}\mu \\&\leqq C\Lambda ^4\int _{\{\gamma >0\}}|A|^2\textrm{d}\mu + C(\lambda _1^2+|\lambda _2|^{\frac{4}{3}}+c_0^4)\int _{\Sigma }|A|^2\gamma ^4\textrm{d}\mu \\&\quad + C c_0^2\int _{\Sigma }|A|^4\gamma ^4\textrm{d}\mu . \end{aligned}$$

The claim follows noting that $c_0^2\int _{\Sigma }|A|^4\gamma ^4\textrm{d}\mu \leqq \varepsilon \int _{\Sigma }|A|^6\gamma ^4\textrm{d}\mu + C(\varepsilon )c_0^4 \int _{\Sigma }|A|^2\gamma ^4\textrm{d}\mu$. □

Still, following the arguments of [29, 47, 48], we introduce the following notation:

### Definition 3.3

Consider a family of immersions $f:[0,T)\times \Sigma \rightarrow {\mathbb {R}}^3$. For t∈[0,T) and r>0, the *curvature concentration function* is defined as

$$\begin{aligned} \kappa (t,r)=\sup _{x\in {\mathbb {R}}^3} \int _{B_r(x)} |A|^2\textrm{d}\mu . \end{aligned}$$

Following the notation of [30], the integrals above have to be understood over the preimage of the open ball $B_r(x)\subset {\mathbb {R}}^3$ under *f*(*t*), that is, the domain of integration is $(f(t))^{-1}(B_r(x))\subset \Sigma$. With this notation, one obtains the following corollary as an integrated version of Proposition 3.2.

### Corollary 3.4

Let $\varepsilon _0\in (0,\infty )$, $\delta _0\in (0,\infty )$ as in Proposition 3.2. There exists a uniform constant C>0 such that, for $A_0,V_0>0$ satisfying (1.9), $c_0\in {\mathbb {R}}$ and an $(A_0,V_0,c_0)$-Helfrich flow $f:[0,T)\times \Sigma \rightarrow {\mathbb {R}}^3$, one has the following: if there exists ρ>0 such that

$$\begin{aligned} \kappa (t,\rho )<\varepsilon _0\quad \hbox { for all}\ t\in [0,T), \end{aligned}$$

then, for all $x\in {\mathbb {R}}^3$ and 0≦t<T,

$$\begin{aligned}&\int _{B_{\frac{\rho }{2}}(x)}|A|^2\textrm{d}\mu \Big |_t + \delta _0\int _{0}^{t}\int _{B_{\frac{\rho }{2}}(x)}\bigl ( |\nabla ^2A|^2+|A|^2|\nabla A|^2+|A|^6 \bigr )\textrm{d}\mu \textrm{d}\tau \\&\quad \leqq \int _{B_{\rho }(x)}|A|^2\textrm{d}\mu \Big |_{t=0} + \frac{C}{\rho ^4} \int _{0}^{t}\int _{B_{\rho }(x)}|A|^2\textrm{d}\mu \textrm{d}\tau \\&\quad \quad + C\int _{0}^{t}\bigl (\lambda _1^2+|\lambda _2|^{\frac{4}{3}}+c_0^4\bigr )\int _{B_{\rho }(x)}|A|^2\textrm{d}\mu \textrm{d}\tau . \end{aligned}$$

### Proof

Choose ${\widetilde{\gamma }}\in C_c^{\infty }({\mathbb {R}}^3)$ with $\chi _{B_{\frac{\rho }{2}}(x)}\leqq {\widetilde{\gamma }}\le \chi _{B_{\rho }(x)}$, $\Vert D{\widetilde{\gamma }}\Vert _{\infty }\leqq C/\rho$ and $\Vert D^2{\widetilde{\gamma }}\Vert _{\infty }\leqq C/\rho ^2$. The claim follows by simply integrating Proposition 3.2 in time with $\Lambda =C/\rho$. □

## A Priori Control of the Lagrange Multipliers

### Lemma 4.1

Consider $A_0,V_0>0$ satisfying $\sigma =36\pi \frac{V_0^2}{A_0^3}\in (0,1)$ and $c_0\in {\mathbb {R}}$. If $f:[0,T)\times \Sigma \rightarrow {\mathbb {R}}^3$ is an $(A_0,V_0,c_0)$-Helfrich flow with

4.1 $$\begin{aligned} \sqrt{{\mathcal {H}}_{c_0}(f_0)}+\frac{c_0}{2}\sqrt{A_0}\le \sqrt{\frac{4\pi }{\sigma }}(1-\eta ) \end{aligned}$$

for some $\eta \in (0,1)$, we find for j=1,2

$$\begin{aligned} |\lambda _j|&\leqq C(\sigma ,\eta ,\operatorname {genus}\Sigma )\frac{(1+\sqrt{c_0^2A_0})^{j-1}}{A_0^{j/2}} \\&\quad \cdot \Bigl (\Bigl [\int _{\Sigma }|\partial _tf|\textrm{d}\mu + |c_0|\Bigl ]\big (1+\sqrt{c_0^2A_0}\big ) + c_0^2\int _{\Sigma }|A|\textrm{d}\mu + \int _{\Sigma }|A|^3\textrm{d}\mu \Bigr ). \end{aligned}$$

### Proof

The assertion is proved by testing the evolution equation (2.7) in two geometrically natural ways which yields a linear system for $\lambda _1$ and $\lambda _2$.

**Affine variation:** Fix any t∈[0,T) and $x_t\in f(t,\Sigma )$. Using (2.7), (2.14) and the scaling properties of area and volume,

4.2 $$\begin{aligned}&-\int _{\Sigma } \langle \partial _tf,f-x_t\rangle \textrm{d}\mu \nonumber \\&\quad = \left. \frac{\textrm{d}}{\textrm{d}\rho }\right| _{\rho =1} \left( 2{\mathcal {H}}_{c_0}(\rho (f-x_t))+\lambda _1(f){\mathcal {A}}(\rho (f-x_t))+\lambda _2(f){\mathcal {V}}(\rho (f-x_t))\right) \nonumber \\&\quad = 2 \left. \frac{\textrm{d}}{\textrm{d}\rho }\right| _{\rho =1}{\mathcal {H}}_{\rho c_0}(f) + 2\lambda _1(f){\mathcal {A}}(f) + 3\lambda _2(f){\mathcal {V}}(f) \nonumber \\&\quad \!=\! \left. \frac{\textrm{d}}{\textrm{d}\rho }\right| _{\rho =1} \Big (2{\mathcal {W}}(f)\!-\!\rho c_0 \int _{\Sigma }H\textrm{d}\mu \!+\!\frac{1}{2}c_0^2\rho ^2{\mathcal {A}}(f)\Big ) \!+\! 2\lambda _1(f){\mathcal {A}}(f) \!+\! 3\lambda _2(f){\mathcal {V}}(f)\nonumber \\&\quad =2\lambda _1(f){\mathcal {A}}(f) + 3\lambda _2(f){\mathcal {V}}(f) - c_0\int _{\Sigma }H\textrm{d}\mu +c_0^2{\mathcal {A}}(f). \end{aligned}$$

Note that one can estimate $|f-x_t|\leqq \textrm{diam}(f)$.

**Variation of the volume:** Multiplying (2.7) by ν yields at time *t*

4.3 $$\begin{aligned} 0&= \partial _t{\mathcal {V}}(f) = -\int _{\Sigma }\langle \partial _t f,\nu \rangle \textrm{d}\mu \nonumber \\&= \int _{\Sigma }|A^0|^2H\textrm{d}\mu -c_0\int _{\Sigma }(|A^0|^2-\frac{1}{2}H(H-c_0))\textrm{d}\mu - \lambda _1\int _{\Sigma }H\textrm{d}\mu - \lambda _2{\mathcal {A}}(f)\nonumber \\&= \int _{\Sigma }|A^0|^2H\textrm{d}\mu +2c_0\int _{\Sigma }K\textrm{d}\mu -(\lambda _1+\frac{1}{2}c_0^2)\int _{\Sigma }H\textrm{d}\mu - \lambda _2{\mathcal {A}}(f)\nonumber \\&=\int _{\Sigma }|A^0|^2H\textrm{d}\mu +c_08\pi (1-\operatorname {genus}\Sigma ) -(\lambda _1+\frac{1}{2}c_0^2)\int _{\Sigma }H\textrm{d}\mu - \lambda _2{\mathcal {A}}(f), \end{aligned}$$

where we used the Gauss–Bonnet theorem. That is, we obtain a linear system for $\lambda _1$ and $\lambda _2$ with coefficient matrix *M* given by

$$\begin{aligned} M=\begin{pmatrix} 2A_0& 3V_0\\ \int _{\Sigma }H\textrm{d}\mu & A_0 \end{pmatrix}. \end{aligned}$$

First, due to (4.1),

$$\begin{aligned} \int _{\Sigma } H\textrm{d}\mu \leqq 2\sqrt{{\mathcal {H}}_{c_0}(f)A_0} + c_0A_0 \leqq 2\sqrt{\frac{4\pi }{\sigma }}\sqrt{A_0}(1-\eta ). \end{aligned}$$

Thus, also using $3V_0=\sqrt{\frac{\sigma }{4\pi }}A_0^{\frac{3}{2}}$, one finds

$$\begin{aligned} \det M&= 2A_0^2-3V_0\int _{\Sigma }H\textrm{d}\mu = 2A_0^2\bigl (1-\sqrt{\frac{\sigma }{4\pi }}\frac{1}{2\sqrt{A_0}}\int _{\Sigma }H\textrm{d}\mu \bigr )\geqq 2A_0^2\eta >0. \end{aligned}$$

Therefore, the solution of the linear system (4.2), (4.3) isNext, we notice that if $c_0\ge 0$, then Simon’s diameter bound [54, Lemma 1.1] and the inequalities (2.4), (4.1) imply

4.4 $$\begin{aligned} |f-x_t|\leqq \textrm{diam}(f) \leqq C\sqrt{A_0{\mathcal {W}}(f)} \leqq C(\sigma ,\eta ) \sqrt{A_0}. \end{aligned}$$

On the other hand, if $c_0<0$, then [51, Theorem 1.2] and the inequality (4.1) imply

4.5 $$\begin{aligned} |f-x_t|\leqq \textrm{diam}(f) \leqq C\sqrt{A_0{\mathcal {H}}_{c_0}(f)} \leqq C(\sigma ,\eta )(1+\sqrt{c_0^2A_0}) \sqrt{A_0}. \end{aligned}$$

For estimating $|\lambda _2|$, we especially useby Cauchy-Schwarz and (4.1). Moreover, again by Cauchy-Schwarz and (4.1),

$$\begin{aligned} \Big |c_0\int _\Sigma (H-c_0)\textrm{d}\mu \Big | \le 2\sqrt{{\mathcal {H}}_{c_0}(f)}\sqrt{A_0} |c_0|\le C(\sigma ,\eta )\big (1+\sqrt{c_0^2A_0}\big )\sqrt{A_0}|c_0|. \end{aligned}$$

Now the claim follows using the inequalities above. □

### Lemma 4.2

There exists a universal constant $0<\varepsilon _1\le \varepsilon _0$ where $\varepsilon _0$ is as in Proposition 3.2 with the following property. In the setting of Lemma 4.1, let ρ>0 be such that

$$\begin{aligned} \kappa (t,\rho )\le \varepsilon <\varepsilon _1\quad \hbox { for all}\ t\in [0,T). \end{aligned}$$

Then, for all t∈[0,T),

4.6 $$\begin{aligned}&\int _0^t\bigl (\lambda _1^2+|\lambda _2|^{\frac{4}{3}}+c_0^4\bigr )\textrm{d}\tau \nonumber \\&\quad \leqq C(\sigma ,\eta ,\operatorname {genus}\Sigma )(1+c_0^2A_0)^{\frac{16}{3}} \Big [{\mathcal {H}}_{c_0}(f(0))-{\mathcal {H}}_{c_0}(f(t)) + \frac{\sqrt{t}}{\rho ^2}\Big (1+\frac{\sqrt{t}}{\rho ^2}\Big )\Big ]. \end{aligned}$$

### Proof

First, summing up the local bounds of Corollary 3.4 as in [47, Proposition 4.2], one obtains

$$\begin{aligned} \int _0^t\int _{\Sigma }|A|^6\textrm{d}\mu \textrm{d}\tau&\le C\int _{\Sigma }|A|^2\textrm{d}\mu \Big |_{t=0} + \frac{C}{\rho ^4}\int _0^t\int _{\Sigma }|A|^2\textrm{d}\mu \textrm{d}\tau \\&\quad + C\int _0^t\bigl (\lambda _1^2+|\lambda _2|^{\frac{4}{3}}+c_0^4\bigr )\int _{\Sigma }|A|^2\textrm{d}\mu \textrm{d}\tau . \end{aligned}$$

Using (4.1), (2.4), and the energy decay Remark 2.4, we estimate

4.7 $$\begin{aligned} \int _{\Sigma }|A|^2\textrm{d}\mu =4{\mathcal {W}}(f)-8\pi (1-\operatorname {genus}\Sigma )\leqq C(\operatorname {genus}\Sigma )(1+c_0^2A_0) \end{aligned}$$

so that

4.8 $$\begin{aligned} \int _0^t\int _{\Sigma }|A|^6\textrm{d}\mu \textrm{d}\tau&\le C(\operatorname {genus}\Sigma )(1+c_0^2A_0)\Bigl ( 1+\frac{t}{\rho ^4} + \int _0^t\bigl (\lambda _1^2+|\lambda _2|^{\frac{4}{3}}+c_0^4\bigr )\textrm{d}\tau \Bigr ). \end{aligned}$$

Using Lemma 4.1 and (4.7), we infer for $C=C(\sigma ,\eta ,\operatorname {genus}\Sigma )$

4.9 $$\begin{aligned} \int _0^t \lambda _1^2\textrm{d}\tau&\le C(1+c_0^2A_0)\Big (\int _0^t\int _{\Sigma }|\partial _tf|^2\textrm{d}\mu \textrm{d}\tau \nonumber \\&\quad + t\frac{c_0^2}{A_0} + tc_0^4 + \frac{1}{A_0}\int _0^t\int _{\Sigma }|A|^4\textrm{d}\mu \textrm{d}\tau \Big ) \nonumber \\&\leqq C (1+c_0^2A_0)\Big (\int _0^t\int _{\Sigma }|\partial _tf|^2\textrm{d}\mu \textrm{d}\tau + t\frac{c_0^2}{A_0}(1+c_0^2A_0) \nonumber \\&\quad + \frac{\sqrt{t(1+c_0^2A_0)}}{A_0}\Big [\int _0^t\int _{\Sigma }|A|^6\textrm{d}\mu \textrm{d}\tau \Big ]^{\frac{1}{2}}\Big ) \end{aligned}$$

Combined with (4.8), one finds

$$\begin{aligned} \int _0^t\lambda _1^2\textrm{d}\tau&\leqq C (1+c_0^2A_0)^2\Bigl ( \int _0^t\int _{\Sigma }|\partial _tf|^2\textrm{d}\mu \textrm{d}\tau + t\frac{c_0^2}{A_0}\\&\quad + \frac{\sqrt{t}}{A_0} \Bigl [1+\frac{\sqrt{t}}{\rho ^2}+\Bigl (\int _0^t\bigl (\lambda _1^2+|\lambda _2|^{\frac{4}{3}}+c_0^4\bigr )\textrm{d}\tau \Bigr )^{\frac{1}{2}}\Bigr ]\Bigr ). \end{aligned}$$

Using again Lemma 4.1, one proceeds similarly for $\int _0^t|\lambda _2|^{\frac{4}{3}}\textrm{d}\tau$ using the following observations. First,

$$\begin{aligned} \frac{1}{A_0^{\frac{4}{3}}} \int _0^t\Bigl (\int _{\Sigma }|\partial _tf|\textrm{d}\mu \Bigr )^{\frac{4}{3}}\textrm{d}\tau&\leqq \frac{1}{A_0^{\frac{4}{3}}} \int _0^t \Bigl (\int _{\Sigma }|\partial _tf|^2\textrm{d}\mu \Bigr )^{\frac{2}{3}} A_0^{\frac{2}{3}}\textrm{d}\tau \\&\le \int _0^t\int _{\Sigma }|\partial _tf|^2\textrm{d}\mu \textrm{d}\tau + C\frac{t}{A_0^2}. \end{aligned}$$

Furthermore,

$$\begin{aligned} \frac{1}{A_0^{\frac{4}{3}}}\int _0^t \Bigl (\int _{\Sigma }|A|^3\textrm{d}\mu \Bigr )^{\frac{4}{3}}\textrm{d}\tau \le \frac{1}{A_0}\int _0^t\int _{\Sigma }|A|^4\textrm{d}\mu \textrm{d}\tau , \end{aligned}$$

a term which had already been estimated in (4.9). Similarly,

$$\begin{aligned} \Big (\frac{|c_0|(1+\sqrt{c_0^2A_0})}{A_0} + \frac{c_0^2}{A_0}\int _\Sigma |A|\textrm{d}\mu \Big )^\frac{4}{3}&\leqq C \Big (\frac{|c_0|(1+\sqrt{c_0^2A_0})}{A_0}\Big )^\frac{4}{3} \\&\quad + C\frac{|c_0|^\frac{8}{3}}{A_0^\frac{2}{3}}\Big (\int _\Sigma |A|^2\textrm{d}\mu \Big )^\frac{2}{3} \\&\leqq C\frac{(1+c_0^2A_0)^2}{A_0^2}. \end{aligned}$$

We thus arrive at

$$\begin{aligned} \int _0^t\bigl (\lambda _1^2 +|\lambda _2|^\frac{4}{3} +c_0^4\bigr )\textrm{d}\tau&\le C\cdot (1+c_0^2A_0)^{\frac{8}{3}}\Big (\int _0^t\int _\Sigma |\partial _tf|^2\textrm{d}\mu \textrm{d}\tau +\frac{t}{A_0^2} \\&\qquad + \frac{\sqrt{t}}{A_0} \Bigl [1+\frac{\sqrt{t}}{\rho ^2}+\Bigl (\int _0^t\bigl (\lambda _1^2+|\lambda _2|^{\frac{4}{3}}+c_0^4\bigr )\textrm{d}\tau \Bigr )^{\frac{1}{2}}\Bigr ]\Big ). \end{aligned}$$

Finally, by Simon’s monotonicity formula, one has the estimate

$$\begin{aligned} \rho ^2\leqq C {\mathcal {A}}(f) = C A_0 \end{aligned}$$

if one takes $\varepsilon _1>0$ sufficiently small, cf. [47, Lemma 4.1]. Altogether, the claim follows from Remark 2.4 after absorbing. □

## Construction of a Blow-Up

### Bounds on Higher-Order Derivatives of the Second Fundamental Form

For the life-span theorem and the construction of a blow-up sequence and a concentration limit, we also need an analogue of Corollary 3.4 for higher-order derivatives of the second fundamental form *A*. This is obtained with similar arguments via local energy estimates, cf. [48, Appendix B]. This section’s main result Proposition 5.2 is an analog of [48, Proposition 3.7] which in turn is based on [29, Theorem 3.5].

#### Proposition 5.1

Let $A_0,V_0>0$ satisfy (1.9), $c_0\in {\mathbb {R}}$, $f:[0,T)\times \Sigma \rightarrow {\mathbb {R}}^3$ be an $(A_0,V_0,c_0)$-Helfrich flow, and consider γ as in (3.1). For $m\in {\mathbb {N}}_0$, $s\ge 2\,m+4$, and $\phi =\nabla ^m A$, one has

$$\begin{aligned}&\frac{\mathrm d}{\mathrm dt}\int _\Sigma |\phi |^2\gamma ^s\textrm{d}\mu \\&\quad \quad + \frac{1}{2}\int _\Sigma |\nabla ^2 \phi |^2\gamma ^s\textrm{d}\mu \le C\big (\lambda _1^2+|\lambda _2|^{\frac{4}{3}}+c_0^4+\Vert A\Vert ^4_{L^\infty (\{\gamma>0\})}\big )\int _\Sigma |\phi |^2\gamma ^s\textrm{d}\mu \\&\qquad + C\big (1+\lambda _1^2+|\lambda _2|^{\frac{4}{3}}+c_0^4+\Vert A\Vert ^4_{L^\infty (\{\gamma>0\})}\big )\int _{\{\gamma >0\}}|A|^2\textrm{d}\mu \end{aligned}$$

for some C=C(s,m,Λ) with Λ as in (3.1).

#### Proof

We will use the operator $P_r^m$ as introduced in [30], cf. [48, Appendix B] and [53, Appendix A]. Writing $\partial _tf=\xi \nu$, the flow equation then reads as

$$\begin{aligned} \xi = -\Delta H + P_3^0(A)+c_0P_2^0(A) + (\lambda _1+\frac{1}{2}c_0^2)P_1^0(A) + \lambda _2. \end{aligned}$$

For $m\in {\mathbb {N}}_0$, a simple proof by induction using Simon’s identity $\nabla ^2\Delta H = \Delta ^2A + P_3^2(A)$ and [30, Lemma 2.3] yields

5.1 $$\begin{aligned}&\partial _t\nabla ^mA + \Delta ^2\nabla ^m A = P_3^{m+2}(A)+P_5^m(A) +c_0\big (P_2^{m+2} + P_4^m(A)\big )\nonumber \\&\quad \quad +\left( \lambda _1+\frac{1}{2}c_0^2\right) \left( P_1^{m+2}(A) + P_3^m(A)\right) + \lambda _2P_2^m(A), \end{aligned}$$

cf. the proof [48, Lemma B.1]. Up to the last term $\lambda _2P_2^m(A)$, we formally recover [53, Equation (A.3)], whereas the exceptional term $\lambda _2P_2^m(A)$ is contained in the analogous equation for the isoperimetric constrained flow [48, Lemma B.2]. Thus, the conclusion follows from [30, Lemma 3.2] by combining the estimates in the proofs [53, Proposition A.1] and [48, Propostition B.3]. □

#### Proposition 5.2

Let $A_0,V_0>0$ satisfy (1.9), $c_0\in {\mathbb {R}}$ and $\varepsilon _0$ as in Proposition 3.2. Further consider an $(A_0,V_0,c_0)$-Helfrich flow $f:[0,T)\times \Sigma \rightarrow {\mathbb {R}}^3$ and suppose that ρ>0 satisfies $T\leqq T^*\rho ^4$ for some $0<T^*<\infty$ and

5.2 $$\begin{aligned} \kappa (t,\rho )\le \varepsilon<\varepsilon _0\quad \hbox { for all}\ 0\leqq t<T. \end{aligned}$$

Further, assume

5.3 $$\begin{aligned} \int _0^T\bigl (\lambda _1^2+|\lambda _2|^{\frac{4}{3}}+c_0^4\bigr )\textrm{d}\tau \le {\overline{L}}<\infty . \end{aligned}$$

For all t∈(0,T) and $m\in {\mathbb {N}}_0$, one has the local estimates

$$\begin{aligned} \Vert \nabla ^m A\Vert _{L^2(B_{\rho /8}(x))}&\leqq C(m,T^*,{\overline{L}})\sqrt{\varepsilon }t^{-\frac{m}{4}},\\ \Vert \nabla ^m A\Vert _{L^{\infty }}&\leqq C(m,T^*,{\overline{L}})\sqrt{\varepsilon }t^{-\frac{m+1}{4}}, \end{aligned}$$

and the full $L^2(\textrm{d}\mu )$-bounds

$$\begin{aligned} \Vert \nabla ^m A\Vert _{L^2(\textrm{d}\mu )}\leqq C(m,T^*,{\overline{L}})t^{-\frac{m}{4}}\Bigl (\int _{\Sigma }|A|^2\textrm{d}\mu \big |_{t=0}\Bigr )^{\frac{1}{2}}. \end{aligned}$$

#### Proof

Firstly, notice that by Lemma 2.11, the time integral of

$$\begin{aligned} L(t):=\lambda _1^2 + |\lambda _2|^{\frac{4}{3}} + c_0^4 \end{aligned}$$

is invariant under parabolic rescaling. Whence it is enough to consider the case ρ=1. Fix $x\in {\mathbb {R}}^3$ and define $K(t):=\int _{B_1(x)}|A|^2\textrm{d}\mu$. For 0<t<T, Equation (5.2) and Corollary 3.4 imply

$$\begin{aligned} \int _0^t\int _{B_{1/2}(x)}|\nabla ^2 A|^2\textrm{d}\mu \textrm{d}\tau \leqq C\Big (K(0) +\int _0^t(1+L)K\textrm{d}\tau \Big ). \end{aligned}$$

By combining the interpolation estimates in [29, Lemma 2.8] and [30, Lemma 4.2], and using (5.3) and $T\leqq T^*$ it follows

$$\begin{aligned} \int _0^t\Vert A\Vert _{L^\infty (B_{1/4}(x))}^4\textrm{d}\tau \leqq C\Big (K(0) +\int _0^t(1+L)K\textrm{d}\tau \Big )\leqq C(T^*,{\bar{L}}). \end{aligned}$$

Summarizing, we have

$$\begin{aligned} \int _0^t\big (1+L+\Vert A\Vert _{L^\infty (B_{1/4}(x))}^4\big )\textrm{d}\tau \leqq C(T^*,{\bar{L}}) \end{aligned}$$

which is [48, (3.18)]. Now, using Proposition 5.1 instead of [48, Proposition B.3], the same proof of [48, Proposition 3.7] applies. □

### Life-Span Result

The fundamental underlying result for the blow-up construction in the subsequent sections is a suitable life-span result: If one has control of the *curvature concentration* and of the nonlocal Lagrange multipliers, then one obtains a (properly scaling, universal) lower bound on the maximal existence time and some control of the curvature concentration forward in time. Such a life-span result was also the starting point of the study of the Willmore flow in [30]. Also see [48, Propositions 5.1 and 5.2] for analogous results in the context of a nonlocal flow.

#### Proposition 5.3

Let $\varepsilon _1>0$ be as in Lemma 4.2. Then there exist universal constants $0<{\overline{\varepsilon }}<\varepsilon _1$ and ${\overline{\delta }}>0$ such that the following is satisfied. Let $A_0,V_0>0$ satisfy (1.9), $c_0\in {\mathbb {R}}$ and suppose that $f_0:\Sigma \rightarrow {\mathbb {R}}^3$ is an embedding with ${\mathcal {V}}(f_0)=V_0$, ${\mathcal {A}}(f_0)=A_0$, and

5.4 $$\begin{aligned} {\mathcal {H}}_{c_0}(f_0) < \Omega (A_0,V_0,c_0), \end{aligned}$$

cf. Definition 2.6. Let $f:[0,T)\times \Sigma \rightarrow {\mathbb {R}}^3$ be a maximal $(A_0,V_0,c_0)$-Helfrich flow with initial datum $f_0$. If

- $$\kappa (0,\rho )\le \varepsilon <{\overline{\varepsilon }}$$ for some ρ>0,
- there exists ${\overline{\omega }}>0$ such that, for any $t_0\in [0,\min \{T,\rho ^4{\overline{\omega }}\}]$ with $\kappa (t,\rho )<\varepsilon _1$ for all $0\leqq t<t_0$, we have $\int _0^{t_0}\bigl (\lambda _1^2+|\lambda _2|^{\frac{4}{3}}+c_0^4\bigr )\textrm{d}\tau \le {\overline{\delta }}$,

then the maximal existence time *T* of *f* satisfies $T>{\hat{c}}\rho ^4$ for some ${\hat{c}}={\hat{c}}({\overline{\omega }})\in (0,1)$,

5.5 $$\begin{aligned} \kappa (t,\rho )\le {\hat{c}}^{-1}\varepsilon \quad \text {for all } t\in \bigl [0,{\hat{c}}\rho ^4\bigr ],\quad \text { and }\quad \int _0^{{\hat{c}}\rho ^4}\bigl (\lambda _1^2+|\lambda _2|^{\frac{4}{3}}+c_0^4\bigr )\textrm{d}\tau \le {\overline{\delta }}. \end{aligned}$$

#### Proof

After scaling as in Lemma 2.11, we may assume without loss of generality that ρ=1. Denoting by Γ>1 the minimal number of balls of radius $\frac{1}{2}$ necessary to cover $B_1(0)\subseteq {\mathbb {R}}^3$, one clearly has

5.6 $$\begin{aligned} \kappa (t,1)\leqq \Gamma \cdot \kappa (t,\frac{1}{2}). \end{aligned}$$

Then choose ${\overline{\varepsilon }}=\frac{\varepsilon _1}{3\Gamma }$. The constant ${\overline{\delta }}$ is to be chosen later.

Note that $\kappa (t):=\kappa (t,1)$ is a continuous function with $\kappa (0)\le \varepsilon <{\overline{\varepsilon }}$. Therefore, for some $\omega \in (0,{\overline{\omega }}]$ to be chosen later,

$$\begin{aligned} t_0:= \sup \{t\in [0,\min \{T,\omega \}]:\kappa \le 3\Gamma \varepsilon \hbox { on}\ [0,t)\} \in (0,\min \{T,\omega \}]. \end{aligned}$$

By the choice of ${\overline{\varepsilon }}$, we have $\kappa (t)\le 3\Gamma \varepsilon < \varepsilon _1$ for $t\in [0,t_0)$. Thus, Corollary 3.4 and (b) yield

$$\begin{aligned} \int _{B_{\frac{1}{2}}(x)}|A|^2\textrm{d}\mu \le \int _{B_1(x)}|A|^2\textrm{d}\mu \Big |_{t=0} + 3 {\widetilde{C}}\Gamma \varepsilon t + 3{\widetilde{C}}\Gamma \varepsilon {\overline{\delta }}, \end{aligned}$$

for all $0\leqq t<t_0$ where ${\widetilde{C}}$ is the universal constant “$\,C\,$” from Corollary 3.4. Choose $\omega =\min \{(6{\widetilde{C}}\Gamma )^{-1},{\overline{\omega }}\}$ and ${\overline{\delta }}=(6{\widetilde{C}}\Gamma )^{-1}$. Then

$$\begin{aligned} \int _{B_{\frac{1}{2}}(x)}|A|^2\textrm{d}\mu \le \int _{B_1(x)}|A|^2\textrm{d}\mu \Big |_{t=0} + \frac{\varepsilon }{2}\frac{1}{\omega }t+\frac{\varepsilon }{2} \le 2\varepsilon \quad \hbox { for all}\ 0\leqq t<t_0. \end{aligned}$$

Using (5.6), if $t_0<\min \{T,\omega \}$, one finds $\kappa (t)\leqq 2\Gamma \varepsilon$ for all $0\leqq t<t_0$. However, by the definition of $t_0$ and continuity of κ, in this case, one also has $\kappa (t_0)=3\Gamma \varepsilon$, a contradiction!

**Assumption:** $t_0=T\le \omega$. Using the specific choices for $t_0$ and ${\overline{\varepsilon }}$ made above, one again obtains $\kappa (t)\leqq 3\Gamma \varepsilon <\varepsilon _1$ for 0≦t<T. Particularly, using $T\le {\overline{\omega }}$, Assumption (b) yields $\int _0^T\bigl (\lambda _1^2+|\lambda _2|^{\frac{4}{3}}+c_0^4\bigr )\textrm{d}\tau \le {\overline{\delta }}$. So Proposition 5.2 implies for 0<ξ<T and $m\in {\mathbb {N}}_0$

$$\begin{aligned} \Vert \nabla ^m A\Vert _{L^{\infty }}&\leqq C(m,{\overline{\omega }},{\overline{\delta }},\xi )\quad \text {and}\\ \Vert \nabla ^m A\Vert _{L^{2}(\textrm{d}\mu )}&\leqq C(m,{\overline{\omega }},{\overline{\delta }},\xi ,\operatorname {genus}\Sigma ,{\mathcal {H}}_{c_0}(f_0),c_0^2A_0) \end{aligned}$$

for $t\in [\xi ,T)$, respectively. Thus, for $t\in [\xi ,T)$, using Proposition 2.10 combined with (5.4) to control the denominators in (2.8), (2.9) as in (2.11),

$$\begin{aligned} |\lambda _1|^2&\leqq C(\operatorname {genus}\Sigma , A_0,V_0,c_0,\Omega (A_0,V_0,c_0)-{\mathcal {H}}_{c_0}(f_0))\ \Vert \nabla {\mathcal {H}}_{c_0}(f)\Vert _{L^2(\textrm{d}\mu )}^2 \\&\leqq C(\operatorname {genus}\Sigma , A_0,V_0,c_0,\Omega (A_0,V_0,c_0)-{\mathcal {H}}_{c_0}(f_0),\xi ,{\overline{\omega }},{\overline{\delta }}) \end{aligned}$$

and similarly also for $\lambda _2$. With the same arguments as in [30, pp. 330 – 332], one deduces that *f*(*t*) converges smoothly to an immersion *f*(*T*) as t↗T. Thus, one can restart the flow at time *T* which contradicts the maximality of *T*. Therefore, $T>\omega =t_0$. Consequently, as observed above for $t< t_0 =\omega <T$, we have $\kappa (t)\le 2\Gamma \varepsilon <\frac{2}{3}\varepsilon _1$. Choosing ${\hat{c}}:=\min \{\omega ,(2\Gamma )^{-1}\}>0$, the estimates in (5.5) hence follow. □

Observe that, for the life-span result Proposition 5.3, we assume some sort of boundedness of the nonlocal Lagrange multipliers. More precisely, the energy threshold (5.4) is needed to bound their denominator, see (2.11) and Proposition 2.10, and the $L^p(\textrm{d}t)$ control assumed in Proposition 5.3(b) is needed in the localized estimates of Corollary 3.4. The following corollary now applies Lemma 4.2 to obtain Proposition 5.3(b) under the second part of the energy assumption in (5.7):

#### Corollary 5.4

Let ${\overline{\varepsilon }}>0$ be as in Proposition 5.3, $A_0,V_0>0$ satisfy (1.9), $c_0\in {\mathbb {R}}$, and $f_0:\Sigma \rightarrow {\mathbb {R}}^3$ be an embedding with ${\mathcal {A}}(f_0)=A_0$, ${\mathcal {V}}(f_0)=V_0$, satisfying

5.7 $$\begin{aligned} \sqrt{{\mathcal {H}}_{c_0}(f_0)} \leqq \min \Bigl \{\sqrt{\Omega (A_0,V_0,c_0)(1-\eta )}, \Bigl (\sqrt{\frac{4\pi }{\sigma }}(1-\eta ) - \frac{c_0}{2}\sqrt{A_0}\Big )\Bigr \} \end{aligned}$$

for some $\eta \in (0,1)$. If $f:[0,T)\times \Sigma \rightarrow {\mathbb {R}}^3$ is a maximal $(A_0,V_0,c_0)$-Helfrich flow starting in $f_0$ with

(i) $\kappa (0,\rho )\le \varepsilon <{\overline{\varepsilon }}$ for some ρ>0 and
(ii) $${\mathcal {H}}_{c_0}(f_0)-\lim _{t\nearrow T}{\mathcal {H}}_{c_0}(f(t))\le {\overline{d}}$$ where $${\overline{d}}={\overline{d}}(\eta ,\sigma ,\operatorname {genus}\Sigma ,c_0^2A_0)>0$$,

then $T>{\hat{c}}\rho ^4$ where ${\hat{c}}={\hat{c}}(\eta ,\sigma ,\operatorname {genus}\Sigma ,c_0^2A_0)\in (0,1)$ and we have the estimates (5.5).

#### Proof

One only needs to check that the assumptions (a) and (b) in Proposition 5.3 are satisfied, for some appropriately chosen ${\overline{d}}$ which will be determined by Lemma 4.2. Clearly, (a) is satisfied by (i). For (b), ${\overline{\omega }}>0$ is to be chosen later and suppose that $0<t_0\le \min \{T,\rho ^4{\overline{\omega }}\}$ satisfies $\kappa (t,\rho )<\varepsilon _1$ for all $0\leqq t < t_0$. By the energy threshold (5.7), Lemma 4.2 applies and (4.6) together with (ii) yields

$$\begin{aligned} \int _0^{t_0} \big (\lambda _1^2+|\lambda _2|^{\frac{4}{3}}+c_0^4\big )\textrm{d}t \leqq C(\eta ,\sigma ,\operatorname {genus}\Sigma ,c_0^2A_0)\big ({\overline{d}} + {\overline{\omega }}^{\frac{1}{2}}+{\overline{\omega }}\big ) \le {\overline{\delta }} \end{aligned}$$

if one takes ${\overline{d}}={\overline{d}}(\eta ,\sigma ,\operatorname {genus}\Sigma ,c_0^2A_0)>0$ and ${\overline{\omega }}={\overline{\omega }}(\eta ,\sigma ,\operatorname {genus}\Sigma ,c_0^2A_0)>0$ sufficiently small. □

### Existence of a Blow-Up and Resulting Concentration Limit

#### Lemma 5.5

Let ${\overline{\varepsilon }}>0$ be as in Proposition 5.3, $A_0,V_0>0$ satisfy (1.9), $c_0\in {\mathbb {R}}$, and let $f_0:\Sigma \rightarrow {\mathbb {R}}^3$ be an embedding with ${\mathcal {V}}(f_0)=V_0$, ${\mathcal {A}}(f_0)=A_0$, satisfying (5.7) for some $\eta \in (0,1)$. Fix ${\hat{c}}={\hat{c}}(\eta ,\sigma ,\operatorname {genus}\Sigma ,c_0^2A_0)$ as in Corollary 5.4 and consider a maximal $(A_0,V_0,c_0)$-Helfrich flow $f:[0,T)\times \Sigma \rightarrow {\mathbb {R}}^3$ starting in $f_0$.

Then there exist times $t_j\nearrow T$, radii $(r_j)_{j\in {\mathbb {N}}}\subseteq (0,\infty )$ and points $(x_j)_{j\in {\mathbb {N}}}\subseteq {\mathbb {R}}^3$ such that each $f_j:[0,(T-t_j)/r_j^4)\times \Sigma \rightarrow {\mathbb {R}}^3$,

$$\begin{aligned} f_j(t,p):=\frac{1}{r_j}\big (f(t_j+r_j^4t,p)-x_j\big ) \end{aligned}$$

is a maximal $((1/r_j^2)A_0,(1/r_j^3)V_0,r_jc_0)$-Helfrich flow, $r_j\leqq C\big (\sqrt{A_0{\mathcal {H}}_{c_0}(f_0)} + \frac{1}{2} (c_0)_+A_0\big )$ for a universal constant C>0 where $(c_0)_+=\max \{c_0,0\}$, and

(i) $$t_j+r_j^4{\hat{c}}<T$$;
(ii) $\kappa _j(t,1)\le {\overline{\varepsilon }}$ for all $0\leqq t\leqq {\hat{c}}$ and $\int _0^{{\hat{c}}}\bigl (\lambda _1(f_j)^2+|\lambda _2(f_j)|^{\frac{4}{3}}+(r_jc_0)^4\bigr )\textrm{d}\tau \le {\overline{\delta }}$;
(iii) $\inf _{j\in {\mathbb {N}}}\int _{B_1(0)}|A_{f_j({\hat{c}})}|^2\textrm{d}\mu _{f_j({\hat{c}})}\ge {{\overline{\alpha }}}$ for some ${{\overline{\alpha }}}>0$ depending only on ${{\overline{\varepsilon }}}$ and ${\hat{c}}$.

#### Proof

The same proof as [46, Lemma 6.6 in Article C] gives the existence of α>0 and radii $(r_t)_{0\le t<T}\subseteq (0,\infty )$ with

5.8 $$\begin{aligned} \alpha<\kappa (t,r_t)<{\hat{c}}{\overline{\varepsilon }}. \end{aligned}$$

Indeed, these $r_t$ satisfy $r_t \leqq \textrm{diam}f(t,\Sigma )$ and thus, arguing as in (4.4) and (4.5),

$$\begin{aligned} r_t&\le C {\left\{ \begin{array}{ll} \sqrt{A_0{\mathcal {W}}(f(t))} & \text {if }c_0\geqq 0\\ \sqrt{A_0{\mathcal {H}}_{c_0}(f(t))}& \text {if }c_0<0 \end{array}\right. } \leqq C\sqrt{A_0}{\left\{ \begin{array}{ll} \sqrt{{\mathcal {H}}_{c_0}(f_0)} + \frac{1}{2}\sqrt{c_0^2 A_0}& \text {if }c_0\geqq 0\\ \sqrt{{\mathcal {H}}_{c_0}(f_0)}& \text {if }c_0<0 \end{array}\right. }\\&= C\big (\sqrt{A_0{\mathcal {H}}_{c_0}(f_0)} + \frac{1}{2} (c_0)_+A_0\big ), \end{aligned}$$

using Remark 2.4 and (2.4) in the case where $c_0\geqq 0$. Moreover, there exists $t_0\in [0,T)$ such that, for $t_0\leqq t<T$,

$$\begin{aligned} {\mathcal {H}}_{c_0}(f(t))-\lim _{\tau \nearrow T}{\mathcal {H}}_{c_0}(f(\tau ))\le {\overline{d}} \end{aligned}$$

with ${\overline{d}}$ as in Corollary 5.4. Since both constants ${{\overline{d}}}$ and ${\hat{c}}$ are invariant under parabolic rescaling and using the uniform energy bound in (5.7), Corollary 5.4 implies $t+r_t^4{\hat{c}} < T$. In particular, Claim (i) will be satisfied. Proceeding as in the proof of [53, Lemma 5.5], one deduces from the upper bound on $r_t$ that there exist $t_0\leqq t_j\nearrow T$ and 0<M<2 with

$$\begin{aligned} r_{t_j+r_j^4{\hat{c}}} \leqq Mr_{t_j}\quad \hbox { for all}\ j\in {\mathbb {N}}. \end{aligned}$$

Write $r_j:=r_{t_j}$. Claim (ii) follows from (5.5). By a covering argument, there is N=N(M)>0 with

$$\begin{aligned} \kappa (t,Mr)\leqq N\kappa (t,r)\quad \text {for all }0\leqq t<T\text { and }r>0. \end{aligned}$$

Then $\kappa (t_j+r_j^4{\hat{c}},r_j)\geqq \frac{1}{N}\kappa (t_j+r_j^4{\hat{c}},Mr_j)\geqq \frac{1}{N}\kappa (t_j+r_j^4{\hat{c}},r_{t_j+r_j^4{\hat{c}}})\geqq \frac{1}{N}\alpha$, using (5.8). Appropriately choosing $x_j\in {\mathbb {R}}^3$ yields (iii). □

Using the *a priori* control of $\lambda _1,\lambda _2$ obtained in Section 4, one finds

#### Proposition 5.6

(Existence and properties of a concentration limit) In the setting of Lemma 5.5, there exists a complete, orientable surface ${\hat{\Sigma }}\ne \emptyset$ without boundary and a proper immersion ${\hat{f}}:{\hat{\Sigma }}\rightarrow {\mathbb {R}}^3$ such that, after passing to a subsequence, $r_j\rightarrow r\in [0,\infty )$ and, for a suitable choice of orientation on ${\hat{\Sigma }}$,

(i) ${\hat{f}}_j:=f_j({\hat{c}})\rightarrow {\hat{f}}$ smoothly on compact subsets after positive reparametrizations;
(ii) $$\int _{{\bar{B}}_1(0)}|{\hat{A}}|^2\textrm{d}{\hat{\mu }}>0$$;
(iii) ${\hat{f}}$ is a constrained ${\mathcal {H}}_{rc_0}$-Helfrich immersion;
(iv) ${\mathcal {A}}({\hat{f}}_j)\rightarrow \infty$ if and only if r=0. In this case, ${\hat{f}}$ is a Willmore immersion.

#### Proof

As $r_j\leqq C\big (\sqrt{A_0{\mathcal {H}}_{c_0}(f_0)} + \frac{1}{2} (c_0)_+A_0\big )$ by Lemma 5.5, after passing to a subsequence, we have $r_j\rightarrow r$ with $0\leqq r<\infty$. Using (ii) in Lemma 5.5 and Proposition 5.2, one finds for $0 < t\leqq {\hat{c}}$ and, for all $m\in {\mathbb {N}}_0$,

5.9 $$\begin{aligned} \Vert \nabla ^m A_j\Vert _{L^{\infty }}&\leqq C(m,{\hat{c}},{\overline{\delta }})t^{-\frac{1}{4}(m+1)}, \end{aligned}$$

5.10 $$\begin{aligned} \Vert \nabla ^m A_j\Vert _{L^{2}(\textrm{d}\mu _j)}&\leqq C(m,{\hat{c}},{\overline{\delta }},\operatorname {genus}\Sigma ,{\mathcal {H}}_{c_0}(f_0),c_0^2A_0)t^{-\frac{m}{4}} \end{aligned}$$

using

$$\begin{aligned} \int _{\Sigma }|A_j|^2\textrm{d}\mu _j\Big |_{t=0}&= 4{\mathcal {W}}(f(t_j)) -4\pi \chi (\Sigma ) \\&\leqq C(\operatorname {genus}\Sigma )(1 + {\mathcal {W}}(f(t_j))) \\&\leqq C(\operatorname {genus}\Sigma )(1+{\mathcal {H}}_{c_0}(f_0) + c_0^2A_0). \end{aligned}$$

Similarly, by Simon’s monotonicity formula [54, Equation (1.3)],

$$\begin{aligned} \mu _{{\hat{f}}_j}(B_R(0))\leqq CR^2{\mathcal {W}}(f(t_j+{\hat{c}} r_j^4))\leqq R^2C({\mathcal {H}}_{c_0}(f_0),c_0^2A_0). \end{aligned}$$

The localized version of Langer’s compactness theorem in [29, Theorem 4.2], also cf. [47, Appendix A], yields the following. There exists a complete 2-manifold ${\hat{\Sigma }}$ without boundary and a proper immersion ${\hat{f}}:{\hat{\Sigma }}\rightarrow {\mathbb {R}}^3$ such that, after passing to a subsequence, ${\hat{f}}_j=f_j({\hat{c}})\rightarrow {\hat{f}}$ smoothly on compact subsets of ${\mathbb {R}}^3$ after reparametrization. That is, writing ${\hat{\Sigma }}(R)=\{p\in {\hat{\Sigma }}\mid |{\hat{f}}(p)|<R\}$ for R>0, one has a representation

5.11 $$\begin{aligned} {\hat{f}}_j\circ \phi _j = {\hat{f}} + u_j\quad \hbox { on}\ {\hat{\Sigma }}(j) \end{aligned}$$

where

$$\begin{aligned}&\phi _j:{\hat{\Sigma }}(j)\rightarrow U_j\subseteq \Sigma \text { is a diffeomorphism},\\&\{|f_j|<R\} \subseteq \phi _j({\hat{\Sigma }}(j))\text { for }j\geqq j(R),\text { for all }R>0,\\&u_j\in C^{\infty }({\hat{\Sigma }}(j),{\mathbb {R}}^3) \text { is normal along }{\hat{f}},\\&\Vert {\hat{\nabla }}^mu_j\Vert _{L^{\infty }({\hat{\Sigma }}(j))}\rightarrow 0\text { for all }m\in {\mathbb {N}}_0. \end{aligned}$$

This already proves (i). Moreover, (ii) simply follows from Lemma 5.5(iii) which also gives ${\hat{\Sigma }}\ne \emptyset$.

Now fix some $0<\xi <{\hat{c}}$ and consider the flows

$$\begin{aligned} {\tilde{f}}_j:[\xi ,{\hat{c}}]\times {\hat{\Sigma }}(j)\rightarrow {\mathbb {R}}^3,\quad {\tilde{f}}_j(t,p)=\frac{1}{r_j}(f(t_j+r_j^4t,\phi _j(p))-x_j). \end{aligned}$$

Particularly, ${\tilde{f}}_j({\hat{c}})={\hat{f}}+u_j$ smoothly converges to ${\hat{f}}$ on compact subsets of ${\hat{\Sigma }}$. Also, the curvature estimates in (5.9) remain valid for the reparametrized flows ${\tilde{f}}_j$ where the powers of $t^{-1}$ are uniformly controlled by powers of $\xi ^{-1}$. In view of Proposition 2.8, we may combine Equations (2.8)–(2.11) with Proposition 2.10 to deduce that for some constant *C* depending on $A_0,V_0,c_0,\eta ,\operatorname {genus}\Sigma$, there holds

5.12 $$\begin{aligned} |\lambda _1(f_j)|&\leqq C \Vert \nabla {\mathcal {H}}_{r_jc_0}({f}_j)\Vert _{L^2(\Sigma ,\textrm{d}{\mu }_j)},\nonumber \\ |\lambda _2({f}_j)|&\leqq \frac{C}{\sqrt{{\mathcal {A}}({f}_j)}} \Vert \nabla {\mathcal {H}}_{r_jc_0}({f}_j)\Vert _{L^2(\Sigma ,\textrm{d}{\mu }_j)} = r_j \frac{C}{\sqrt{A_0}} \Vert \nabla {\mathcal {H}}_{r_jc_0}({f}_j)\Vert _{L^2(\Sigma ,\textrm{d}{\mu }_j)}. \end{aligned}$$

Using (5.9) as well as $r_j\leqq C\big (\sqrt{A_0{\mathcal {H}}_{c_0}(f_0)} + \frac{1}{2} (c_0)_+A_0\big )$, it follows

5.13 $$\begin{aligned} |\lambda _1(f_j)|+|\lambda _2(f_j)|\leqq C({\hat{c}},{\overline{\delta }},\operatorname {genus}\Sigma ,\xi ,c_0,A_0,V_0,\eta ). \end{aligned}$$

Notice that by the same proof, (5.1) remains valid if on the left hand side, the second fundamental form *A* is replaced by the mean curvature *H*. Combining this with (5.9), (5.10), (5.13), one finds for $\xi \leqq t\leqq {\hat{c}}$ that

$$\begin{aligned} \Vert \partial _t \nabla ^m A_j\Vert _{L^{\infty }}+\Vert \partial _t \nabla ^m A_j\Vert _{L^{2}(\textrm{d}\mu _j)}&\leqq C,\\ \Vert \partial _t \nabla ^m H_j\Vert _{L^{\infty }} +\Vert \partial _t \nabla ^m H_j\Vert _{L^{2}(\textrm{d}\mu _j)}&\leqq C \end{aligned}$$

for a constant $C=C(m,{\hat{c}},{\overline{\delta }},\operatorname {genus}\Sigma ,\xi ,c_0,A_0,V_0,\eta )$. Therefore, using (2.8), (2.9), (2.11), Proposition 2.10, and (5.9), (5.10), we infer for $\xi \leqq t\le {\hat{c}}$

$$\begin{aligned} |\partial _t \lambda _1({f}_j)| +|\partial _t \lambda _2({f}_j)| \leqq C. \end{aligned}$$

for a constant $C=C(m,{\hat{c}},{\overline{\delta }},\operatorname {genus}\Sigma ,\xi ,c_0,A_0,V_0,\eta )$. Therefore, arguing as in [46, Article C, pp. 25–26], after passing to a subsequence, the flows $({\tilde{f}}_j)_{j\in {\mathbb {N}}}$ converge in $C^1([\xi ,{\hat{c}}],C^{\infty }(P))$ for any compact $P\subseteq {\hat{\Sigma }}$ to a limiting flow ${\tilde{f}}_{\textrm{lim}}:[\xi ,{\hat{c}}]\times {\hat{\Sigma }}\rightarrow {\mathbb {R}}^3$. With similar arguments, for the unit-normal fields, one may assume $\nu _j(t,\phi _j(x)) \rightarrow {\tilde{\nu }}_{\textrm{lim}}(t,x)$ in $C^1([\xi ,{\hat{c}}],C^{\infty }(P))$ where ${\tilde{\nu }}_{\textrm{lim}}(t,\cdot )$ is a smooth unit normal field along ${\tilde{f}}_{\textrm{lim}}(t)$. Particularly, ${\hat{\Sigma }}$ is again orientable and we can fix an orientation on ${\hat{\Sigma }}$ such that ${\tilde{\nu }}_{\textrm{lim}}$ is induced by ${\tilde{f}}_{\textrm{lim}}$ via (2.1). In particular, each $\phi _j:{\hat{\Sigma }}(j)\rightarrow U_j\subseteq \Sigma$ is positive for the induced orientations on ${\hat{\Sigma }}(j)\subseteq {\hat{\Sigma }}$ and $U_j\subseteq \Sigma$, respectively. Further, we may assume that

$$\begin{aligned} \lambda _1({f}_j) \rightarrow \lambda _{1,\textrm{lim}}\quad \text {and}\quad \lambda _2({f}_j)\rightarrow \lambda _{2,\textrm{lim}}\quad \text {in } C^0([\xi ,{\hat{c}}],{\mathbb {R}}). \end{aligned}$$

Then, for any compact $P\subseteq {\hat{\Sigma }}$,

$$\begin{aligned} \int _{\xi }^{{\hat{c}}} \int _P |\partial _t{\tilde{f}}_{\lim }|^2\textrm{d}{\tilde{\mu }}_{\lim }\textrm{d}t&= \lim _{j\rightarrow \infty } \int _{\xi }^{{\hat{c}}} \int _P |\partial _t{\tilde{f}}_j|^2\textrm{d}{\tilde{\mu }}_j\textrm{d}t \leqq \lim _{j\rightarrow \infty } \int _{\xi }^{{\hat{c}}} \int _{\Sigma } |\partial _t f_j|^2\textrm{d}\mu _j\textrm{d}t\\&= 2\lim _{j\rightarrow \infty } \big ({\mathcal {H}}_{r_jc_0}(f_j(\xi ))-{\mathcal {H}}_{r_jc_0}(f_j({\hat{c}}))\big ) \\&= 2\lim _{j\rightarrow \infty } \big ({\mathcal {H}}_{c_0}(f(t_j+r_j^4\xi ))-{\mathcal {H}}_{c_0}(f(t_j+r_j^4{\hat{c}}))\big ) = 0, \end{aligned}$$

using Remark 2.4, (2.14), and the smooth convergence established above. So ${\tilde{f}}_{\lim }(t)={\tilde{f}}_{\lim }({\hat{c}}) = {\hat{f}}$ on ${\hat{\Sigma }}$ for all $\xi \leqq t\leqq {\hat{c}}$. Setting ${\hat{\lambda }}_1=\lambda _{1,\lim }({\hat{c}})$, ${\hat{\lambda }}_2=\lambda _{2,\lim }({\hat{c}})$, ${\hat{\nu }}={\tilde{\nu }}_{\lim }({\hat{c}})$, the fact that, due to Lemma 2.11 and the choice of orientation on ${\hat{\Sigma }}$, each ${\tilde{f}}_j$ is an $(r_j^{-2}A_0,r_j^{-3}V_0,r_jc_0)$-Helfrich flow, and $r_j\rightarrow r$ yield with the above convergence in $C^1([\xi ,{\hat{c}}],C^{\infty }(P))$ for any compact $P\subseteq {\hat{\Sigma }}$

$$\begin{aligned}&\big (\Delta {\hat{H}} + |{\hat{A}}^0|^2{\hat{H}} -rc_0(|{\hat{A}}^0|^2-\frac{1}{2}{\hat{H}}^2) -({\hat{\lambda }}_1+\frac{1}{2}r^2c_0^2){\hat{H}} - {\hat{\lambda }}_2\big ){\hat{\nu }}\\&\quad =-\lim _{j\rightarrow \infty } \partial _t{\tilde{f}}_j({\hat{c}}) = -\partial _t{\tilde{f}}_{\lim }({\hat{c}}) = 0\quad \text {on }{\hat{\Sigma }}. \end{aligned}$$

So ${\hat{f}}$ is a constrained ${\mathcal {H}}_{rc_0}$-Helfrich immersion. This proves Claim (iii).

For (iv), since

$$\begin{aligned} {\mathcal {A}}({\hat{f}}_j) = {\mathcal {A}}\Big (\frac{1}{r_j}\big (f(t_j+r_j^4{\hat{c}})-x_j\big )\Big ) = \frac{A_0}{r_j^2}, \end{aligned}$$

one finds r=0 if and only if ${\mathcal {A}}({\hat{f}}_j)\rightarrow \infty$. With (iii), we only need to show that, in this case, ${\hat{\lambda }}_1={\hat{\lambda }}_2=0$. To this end, suppose that r=0 or, equivalently, ${\mathcal {A}}({\hat{f}}_j)\rightarrow \infty$. By Lemma 4.1 and Remark 2.4, for all $j\in {\mathbb {N}}$ and $\xi \in (0,{\hat{c}})$,

$$\begin{aligned}&\int _{\xi }^{{\hat{c}}} |\lambda _1(f_j)|^2\textrm{d}t\leqq C(\sigma ,\eta ,\operatorname {genus}\Sigma )(1+c_0^2A_0) \Big ({\mathcal {H}}_{c_0}(t_j+r_j^4\xi )-{\mathcal {H}}_{c_0}(t_j+r_j^4{\hat{c}}) \\&\quad \quad + \frac{1}{\frac{1}{r_j^2}A_0}\int _{\xi }^{{\hat{c}}}\Big [r_j|c_0| + r_j^2c_0^2\int _{\Sigma }|A_j|\textrm{d}\mu _j + \int _{\Sigma }|A_j|^3\textrm{d}\mu _j \Big ]^2\textrm{d}t\Big ) \rightarrow 0 \end{aligned}$$

as $j\rightarrow \infty$, using (5.9),(5.10), and $r_j\rightarrow 0$. So ${\hat{\lambda }}_1=\lim _{j\rightarrow \infty }\lambda _1(f_j({\hat{c}}))=0$. Furthermore, as in (5.12), one finds

$$\begin{aligned} |\lambda _2({f}_j)|\leqq \frac{C(\sigma )}{\sqrt{{\mathcal {A}}({f}_j)}} \Vert \nabla {\mathcal {H}}_{r_jc_0}({f}_j)\Vert _{L^2(\Sigma ,\textrm{d}{\mu }_j)} = r_j \frac{C(\sigma )}{\sqrt{A_0}} \Vert \nabla {\mathcal {H}}_{r_jc_0}({f}_j)\Vert _{L^2(\Sigma ,\textrm{d}{\mu }_j)} \end{aligned}$$

and $\Vert \nabla {\mathcal {H}}_{r_jc_0}({f}_j)\Vert _{L^2(\Sigma ,\textrm{d}{\mu }_j)}$ is uniformly bounded in $j\in {\mathbb {N}}$ for $\xi \leqq t\leqq {\hat{c}}$ by (5.9)–(5.10). Therefore, we conclude ${\hat{\lambda }}_2=\lim _{j\rightarrow \infty }\lambda _2(f_j({\hat{c}}))=0$. □

## Stability via a Constrained Łojasiewicz–Simon Inequality

In this section, we prove global existence for initial data sufficiently close to a local minimizer. Our method is based on a Łojasiewicz–Simon gradient inequality and the arguments in [14] for the Willmore flow. A version of this inequality for the constrained Canham–Helfrich energy has already been formally derived in a model for fluid vesicle dynamics, see [32, Theorem 1.4]. However, this approach works in a different Sobolev regime and requires a graphical representation close to an embedded surface, thus it is not applicable in our context. Instead, as in [48], we use the sufficient conditions of [45].

### Lemma 6.1

Let $f:\Sigma \rightarrow {\mathbb {R}}^3$ be a constrained ${\mathcal {H}}_{c_0}$-Helfrich immersion with $H_f\not \equiv const$. Then there exist C,ρ>0 and $\theta \in (0,\frac{1}{2}]$ such that for all immersions $h\in W^{4,2}(\Sigma ;{\mathbb {R}}^3)$ with $\Vert h-f\Vert _{W^{4,2}}\leqq \rho$, ${\mathcal {A}}(h)={\mathcal {A}}(f)$, and ${\mathcal {V}}(h)={\mathcal {V}}(f)$ we have

$$\begin{aligned} |{\mathcal {H}}_{c_0}(h)-{\mathcal {H}}_{c_0}(f)|^{1-\theta } \leqq C \Vert 2\nabla {\mathcal {H}}_{c_0}(h) -\lambda _1(h) H_h\nu _h - \lambda _2(h) \nu _h\Vert _{L^2(\textrm{d}\mu _h)}, \end{aligned}$$

where $\lambda _1(h),\lambda _2(h)$ are as in (2.8) and (2.9).

We will not prove this lemma here, since it can be deduced with minor modifications from the case of the Willmore energy with constrained isoperimetric ratio in [48, Section 5.3]. Indeed, the energy ${\mathcal {H}}_{c_0}$ is essentially a lower order perturbation of the Willmore functional, so the necessary Fredholm properties can be deduced from the second variation of ${\mathcal {W}}$ computed in [14]. It remains to check the conditions on the constraints in [45, Corollary 5.2]. Since the constraints on the area and the volume are of lower order, compared to the energy ${\mathcal {H}}_{c_0}$, condition (v) in [45, Corollary 5.2] follows from the representation of the $L^2$-gradients in Proposition 2.3. By a direct computation, the constraints are nondegenerate, that is, assumption (vi) of [45, Corollary 5.2] is satisfied, if $H_f\not \equiv const$.

As a consequence of Lemma 6.1, we find the following asymptotic stability result.

### Lemma 6.2

Let ${\hat{f}}:\Sigma \rightarrow {\mathbb {R}}^3$ be a constrained ${\mathcal {H}}_{c_0}$-Helfrich immersion with ${\mathcal {A}}({\hat{f}})=A_0$, ${\mathcal {V}}({\hat{f}})=V_0$, and $H_{{\hat{f}}}\not \equiv const$. Let $\alpha \in (0,1)$, $k\in {\mathbb {N}}$ with k≧4, and δ>0. Then there exists $\varepsilon =\varepsilon ({\hat{f}},\alpha ,k,\delta )>0$ such that if $f:[0,T)\times \Sigma \rightarrow {\mathbb {R}}^3$ is an $(A_0,V_0,c_0)$-Helfrich flow satisfying

(i) $$\Vert {f_0-{\hat{f}}}\Vert _{C^{k,\alpha }}<\varepsilon $$;
(ii) ${{\mathcal {H}}_{c_0}}(f(t))\geqq {{\mathcal {H}}_{c_0}}({\hat{f}})$ whenever $\Vert {f(t)\circ \Phi (t) - {\hat{f}}}\Vert _{C^k}\leqq \delta$, for some positive diffeomorphisms $\Phi (t) :\Sigma \rightarrow \Sigma$;

then the flow exists globally, that is, we may take T=∞. Moreover, as $t\rightarrow \infty$, it converges smoothly after reparametrization by some positive diffeomorphisms ${\tilde{\Phi }}(t):\Sigma \rightarrow \Sigma$ to a constrained ${\mathcal {H}}_{c_0}$-Helfrich immersion $f_\infty$, satisfying ${{\mathcal {H}}_{c_0}}(f_\infty )={{\mathcal {H}}_{c_0}}({\hat{f}})$ and $\Vert {f_{\infty }-{\hat{f}}}\Vert _{C^k}\le \delta$.

This is exactly the counterpart to [14, Lemma 4.1], [47, Lemma 7.9], and [48, Lemma 5.8] for our gradient flow situation. As a consequence, r>0 in Proposition 5.6 implies convergence.

### Lemma 6.3

Suppose that ${\hat{f}}:{\hat{\Sigma }}\rightarrow {\mathbb {R}}^3$ is a concentration limit as in Proposition 5.6 of a maximal $(A_0,V_0,c_0)$-Helfrich flow $f:[0,T)\times \Sigma \rightarrow {\mathbb {R}}^3$. If r>0, then ${\hat{\Sigma }}\cong \Sigma$ is compact. Further, if ${H}_{{\hat{f}}}\not \equiv const$, then the flow exists for all times, that is, T=∞, and, as $t\rightarrow \infty$, converges smoothly after positive reparametrizations to a constrained ${\mathcal {H}}_{c_0}$-Helfrich immersion $f_\infty$.

### Proof

By Proposition 5.6(iv), r>0 implies $\sup _j {\mathcal {A}}({\hat{f}}_j)<\infty$. Simon’s diameter estimate [54, Lemma 1.1] then implies that ${\hat{f}}({\hat{\Sigma }})$ is compact, and consequently, by [29, Lemma 4.3], we have that ${\hat{\Sigma }}\cong \Sigma$ is compact. In fact, [29, Lemma 4.3] yields that, in (5.11), one may without loss of generality arrange that ${\hat{\Sigma }}(j)={\hat{\Sigma }}$ and $U_j=\Sigma$ such that $\phi _j:{\hat{\Sigma }}\rightarrow \Sigma$ is a positive diffeomorphism for all $j\in {\mathbb {N}}$, after passing to a subsequence. Using (5.11),

$$\begin{aligned} f(t_j+{\hat{c}}r_j^4,\phi _j\circ \phi _1^{-1}(\cdot ))-x_j = r_j {\hat{f}}_j\circ (\phi _j\circ \phi _1^{-1}) \rightarrow r {\hat{f}}\circ \phi _1^{-1}\qquad \text {smoothly on }\Sigma . \end{aligned}$$

Moreover, $\phi _j\circ \phi _1^{-1}:\Sigma \rightarrow \Sigma$ is positive for each $j\in {\mathbb {N}}$. By Proposition 5.6(iii) and (2.14), we have that $r{\hat{f}}\circ \phi _1^{-1}$ is a constrained ${\mathcal {H}}_{c_0}$-Helfrich immersion since $\phi _1^{-1}:\Sigma \rightarrow {\hat{\Sigma }}$ is positive. Furthermore, $H_{r{\hat{f}}\circ \phi _1^{-1}} = r^{-1}H_{{\hat{f}}\circ \phi _1^{-1}} = r^{-1}H_{{\hat{f}}}\circ \phi _1^{-1} \not \equiv const$, so that for *j* large enough, the assumptions of Lemma 6.2 are satisfied and the second statement follows. □

## Convergence for Spherical Immersions

In view of Lemma 6.3, we want to exclude the case r=0 in Proposition 5.6, that is, the case of a noncompact concentration limit. To this end, we proceed by contradiction and first deduce some properties of a noncompact concentration limit by adapting arguments in [31, Appendix A] to the monotonicity formula for the Helfrich energy ${\mathcal {H}}_{c_0}$ found in [43]. In the following, we define the function which is the ${\mathcal {H}}_{c_0}$-analog to [31, Equation (A.4)].

### Definition 7.1

For an immersion $h:\Sigma \rightarrow {\mathbb {R}}^3$ of an oriented, complete surface Σ without boundary and $c_0\in {\mathbb {R}}$, ρ>0, define that

$$\begin{aligned} \gamma _{h,c_0}(\rho )&= \frac{\mu ({\bar{B}}_{\rho }(0))}{\rho ^2} + \frac{1}{16}\int _{{\bar{B}}_{\rho }(0)}(H-c_0)^2\textrm{d}\mu - \frac{c_0}{2} \int _{{\bar{B}}_{\rho }(0)} \frac{\langle h,\nu \rangle }{|h|^2}\textrm{d}\mu \\&\quad + \frac{1}{2\rho ^2} \int _{{\bar{B}}_{\rho }(0)} \langle h,\nu \rangle H\textrm{d}\mu . \end{aligned}$$

### Remark 7.2

By computations in [43, Lemma 4.1] (see also [43, Equation (4.12)]), the mapping $(0,\infty )\ni \rho \mapsto \gamma _{h,c_0}(\rho )$ is nondecreasing for any $c_0\in {\mathbb {R}}$. Moreover, for r>0, one computes that

$$\begin{aligned} \gamma _{r h,r^{-1}c_0}(r\rho ) = \gamma _{h,c_0}(\rho ). \end{aligned}$$

With the monotonicity property and scaling behavior in Remark 7.2, employing arguments similar to [31, Equation (A.23)], one finds the following relation between the density at infinity and the Willmore energy of a noncompact concentration limit.

### Lemma 7.3

Let $A_0,V_0>0$ satisfy (1.9), $c_0\in {\mathbb {R}}$, and consider a sequence of embeddings $f_j:\Sigma \rightarrow {\mathbb {R}}^3$ of a compact, closed and oriented surface Σ with ${\mathcal {A}}(f_j)=A_0$, ${\mathcal {V}}(f_j)=V_0$ and

$$\begin{aligned} \sup _{j\in {\mathbb {N}}} {\mathcal {H}}_{c_0}(f_j) < \Omega (A_0,V_0,c_0). \end{aligned}$$

Suppose that, for $r_j\searrow 0$, ${\hat{f}}:{\hat{\Sigma }}\rightarrow {\mathbb {R}}^3$ is an immersion such that $\frac{1}{r_j}f_j\rightarrow {\hat{f}}$ smoothly on compact subsets of ${\mathbb {R}}^3$ after reparametrization. Then

$$\begin{aligned} \lim _{\rho \rightarrow \infty } \frac{{\hat{\mu }}({\bar{B}}_{\rho }(0))}{\pi \rho ^2} + \frac{1}{4\pi } {\mathcal {W}}({\hat{f}}) < 2. \end{aligned}$$

### Proof

By smooth convergence on compact subsets of ${\mathbb {R}}^3$, one finds

$$\begin{aligned} \gamma _{{\hat{f}},0}(\rho ) = \lim _{j\rightarrow \infty } \gamma _{\frac{1}{r_j}f_j,r_jc_1}(\rho ) \end{aligned}$$

for any $c_1\in {\mathbb {R}}$. Therefore, using Remark 7.2 we have

7.1 $$\begin{aligned} \lim _{\rho \rightarrow \infty } \gamma _{{\hat{f}},0}(\rho )&= \sup _{\rho>0} \gamma _{{\hat{f}},0}(\rho ) = \sup _{\rho>0} \liminf _{j\rightarrow \infty }\gamma _{\frac{1}{r_j}f_j,r_jc_1}(\rho ) \nonumber \\&\leqq \liminf _{j\rightarrow \infty } \sup _{\rho >0} \gamma _{\frac{1}{r_j}f_j,r_jc_1}(\rho ) = \liminf _{j\rightarrow \infty } \lim _{\rho \rightarrow \infty } \gamma _{\frac{1}{r_j}f_j,r_jc_1}(\rho ) \nonumber \\&= \liminf _{j\rightarrow \infty } \lim _{\rho \rightarrow \infty } \gamma _{f_j,c_1}(r_j\rho ) = \liminf _{j\rightarrow \infty } \Big ( \frac{1}{4}{\mathcal {H}}_{c_1}(f_j) - \frac{c_1}{2}\int _{\Sigma } \frac{\langle f_j,\nu _j\rangle }{|f_j|^2}\textrm{d}\mu _j \Big ). \end{aligned}$$

Now, if $c_0\geqq 0$ and $\Omega (A_0,V_0,c_0)= (\sqrt{8\pi }-\frac{1}{2}\sqrt{c_0^2A_0})_+^2$, we may use (7.1) with $c_1=0$ and (2.12) to conclude that

7.2 $$\begin{aligned} \lim _{\rho \rightarrow \infty } \gamma _{{\hat{f}},0}(\rho ) <2\pi . \end{aligned}$$

Otherwise, we use (7.1) with $c_1=c_0$ and (2.13) to conclude (7.2). By the smooth convergence $\frac{1}{r_j}f_j\rightarrow {\hat{f}}$ on compact sets, one finds that

$$\begin{aligned} \frac{1}{4}\int _{{B}_{\rho }(0)} {\hat{H}}^2\textrm{d}{\hat{\mu }}&\le \liminf _{j\rightarrow \infty } \frac{1}{4}\int _{{\bar{B}}_{\rho }(0)} (H_{\frac{1}{r_j}f_j}-r_jc_0)^2\textrm{d}\mu _{\frac{1}{r_j}f_j} \le \liminf _{j\rightarrow \infty } {\mathcal {H}}_{r_jc_0}(\frac{1}{r_j}f_j) \\&= \liminf _{j\rightarrow \infty }{\mathcal {H}}_{c_0}(f_j) < \Omega (A_0,V_0,c_0). \end{aligned}$$

Letting $\rho \rightarrow \infty$, ${\mathcal {W}}({\hat{f}})<\infty$. Therefore, arguing as in [31, Equation (A.14)] yields

$$\begin{aligned} \lim _{\rho \rightarrow \infty } \gamma _{{\hat{f}},0}(\rho ) = \lim _{\rho \rightarrow \infty } \frac{{\hat{\mu }}({\bar{B}}_{\rho }(0))}{\rho ^2} + \frac{1}{4}{\mathcal {W}}({\hat{f}}), \end{aligned}$$

and thus the claim. □

### Lemma 7.4

Let $A_0,V_0>0$ satisfy (1.9), $c_0\in {\mathbb {R}}$, and consider a sequence of spherical embeddings $f_j:{\mathbb {S}}^2\rightarrow {\mathbb {R}}^3$ with ${\mathcal {A}}(f_j)=A_0$, ${\mathcal {V}}(f_j)=V_0$ and

7.3 $$\begin{aligned} \sup _{j\in {\mathbb {N}}} {\mathcal {H}}_{c_0}(f_j) < \Omega (A_0,V_0,c_0). \end{aligned}$$

Suppose that ${\hat{f}}:{\hat{\Sigma }}\rightarrow {\mathbb {R}}^3$ is a Willmore immersion such that, for some $r_j>0$ with $r_j\rightarrow r\in [0,\infty )$, $\frac{1}{r_j}f_j\rightarrow {\hat{f}}$ smoothly on compact subsets of ${\mathbb {R}}^3$ after reparametrization. If $\int _{B_1(0)}|{\hat{A}}|^2\textrm{d}{\hat{\mu }}>0$, then r>0.

### Proof

For the sake of contradiction, suppose that r=0. Lemma 7.3 yields that

$$\begin{aligned} \lim _{\rho \rightarrow \infty } \frac{{\hat{\mu }}({\bar{B}}_{\rho }(0))}{\pi \rho ^2} + \frac{1}{4\pi } {\mathcal {W}}({\hat{f}}) < 2. \end{aligned}$$

Since ${\mathcal {A}}(\frac{1}{r_j}f_j)=\frac{1}{r_j^2}A_0\rightarrow \infty$, one finds $\lim _{\rho \rightarrow \infty } \frac{{\hat{\mu }}({\bar{B}}_{\rho }(0))}{\pi \rho ^2}\geqq 1$, cf. [31, Equation (A.22)]. Particularly, ${\mathcal {W}}({\hat{f}})<4\pi$. Moreover, by smooth convergence on compact subsets, we find that

$$\begin{aligned} \int _{{\hat{\Sigma }}}|{\hat{A}}|^2\textrm{d}{\hat{\mu }}\leqq \liminf _{j\rightarrow \infty } \int _{{\mathbb {S}}^2} |A_{f_j}|^2\textrm{d}\mu _{f_j} \le C(A_0,V_0,c_0)<\infty , \end{aligned}$$

using Gauss–Bonnet, Lemma 2.1, and (7.3). If $x_0\notin {\hat{f}}({\hat{\Sigma }})$, consider the inversion $I(x)=|x-x_0|^{-2}(x-x_0)$ and set ${\overline{\Sigma }}=I({\hat{f}}({\hat{\Sigma }}))\cup \{0\}$. By [31, Lemma 4.3], ${\overline{\Sigma }}$ is a smooth Willmore surface with

$$\begin{aligned} {\mathcal {W}}({\overline{\Sigma }}) = {\mathcal {W}}({\hat{f}}) + 4\pi < 8\pi . \end{aligned}$$

Arguing as on [31, p. 346], since all $f_j$ are spherical immersions, one finds that ${\overline{\Sigma }}$ necessarily also is a (topological) sphere. Thus, by [11, Theorem E and the Remark on p. 47], $\overline{{\Sigma }}$ is a round sphere which in turn yields that ${\hat{f}}({\hat{\Sigma }})$ parametrizes a flat plane. This contradicts $\int _{B_1(0)}|{\hat{A}}|^2\textrm{d}{\hat{\mu }}>0$. □

### Proof of Theorem 1.1.

Using (2.10), either $\partial _tf\equiv 0$ on $[0,T)\times {\mathbb {S}}^2$ or ${\mathcal {H}}_{c_0}(f(t))<{\mathcal {H}}_{c_0}(f_0)$ for t∈(0,T). Thus, replacing $f_0$ by f(δ) for some $\delta \in (0,T)$, we may without loss of generality assume (5.7) for some $\eta \in (0,1)$. Proposition 5.6 thus implies the existence of a concentration limit ${\hat{f}}:{\hat{\Sigma }}\rightarrow {\mathbb {R}}^2$. From Lemma 7.4 we infer r>0. Moreover, by Aleksandrov’s theorem [2] and Assumption (1.9) we have $H_{{\hat{f}}}\not \equiv const$. Hence, the conclusion follows from Lemma 6.3. □

## Convergence for Axisymmetric Tori

For an immersion $\gamma :{\mathbb {S}}^1\rightarrow {\mathbb {H}}^2$ into the hyperbolic plane ${\mathbb {H}}^2={\mathbb {R}}\times (0,\infty )$, consider the associated axisymmetric torus $f_{\gamma }:{\mathbb {T}}^2\rightarrow {\mathbb {R}}^3$ given by

$$\begin{aligned} f_{\gamma }(u,v)=(\gamma ^{(1)}(u),\gamma ^{(2)}(u)\cos (v),\gamma ^{(2)}(u)\sin (v)), \end{aligned}$$

where ${\mathbb {T}}^2={\mathbb {S}}^1\times {\mathbb {S}}^1$ with the usual identification ${\mathbb {S}}^1={{\mathbb {R}}\ }/{2\pi {\mathbb {Z}}}$.

For the $(A_0,V_0,c_0)$-Helfrich flow starting in an axisymmetric torus, one has the following analog of [19, Lemma 3.3] with essentially the same proof with some minor modifications which we therefore postpone to Appendix B.

### Lemma 8.1

Let $f_0=f_{\gamma _0}$ be an axisymmetric torus, $A_0,V_0>0$ satisfy (1.9) and let $c_0\in {\mathbb {R}}$. If $f:[0,T)\times {\mathbb {T}}^2\rightarrow {\mathbb {R}}^3$ is an $(A_0,V_0,c_0)$-Helfrich flow starting in $f_0$, then $f(t)=f_{\gamma (t)}$ for a smooth family of immersions $\gamma :[0,T)\times {\mathbb {S}}^1\rightarrow {\mathbb {H}}^2$.

As in [19], an important quantity is the so-called *hyperbolic length* of γ, that is

8.1 $$\begin{aligned} {\mathcal {L}}_{{\mathbb {H}}^2}(\gamma )=\int _{{\mathbb {S}}^1}|\partial _u\gamma |_{g_{{\mathbb {H}}^2}}\textrm{d}u = \int _{{\mathbb {S}}^1} 1\textrm{d}s \end{aligned}$$

where $\langle \cdot ,\cdot \rangle _{g_{{\mathbb {H}}^2}(p)}=\frac{1}{(p^{(2)})^2}\langle \cdot ,\cdot \rangle _{{\mathbb {R}}^2}$ for $p=(p^{(1)},p^{(2)})\in {\mathbb {H}}^2$ and where $\textrm{d}s=|\partial _u\gamma |_{g_{{\mathbb {H}}^2}}\textrm{d}u$ is the (hyperbolic) arc-length element. In [19], the result in [38] is used to control the hyperbolic length of the profile curves along the Willmore flow: For each ε>0, there exists C=C(ε)>0 such that, if ${\mathcal {W}}(f_{\gamma })\leqq 8\pi -\varepsilon$, then ${\mathcal {L}}_{{{\mathbb {H}}^2}}(\gamma )\leqq C(\varepsilon )$. While such a priori energy control is natural for the Willmore flow considered in [19], it does not immediately apply along an $(A_0,V_0,c_0)$-Helfrich flow starting in an axisymmetric torus. Therefore, in the following proposition, boundedness of the hyperbolic length of the profile curves along the evolution is shown using the Li–Yau inequality in Proposition 2.9, cf. [43, Corollary 4.11], essentially following the strategy in [52, Proposition 4.2].

### Proposition 8.2

Let $f_0=f_{\gamma _0}$ be an embedded axisymmetric torus, let $A_0,V_0>0$ satisfy (1.9), $c_0\in {\mathbb {R}}$ and suppose that

$$\begin{aligned} {\mathcal {H}}_{c_0}(f_0) < \Omega (A_0,V_0,c_0). \end{aligned}$$

If $f:[0,T)\times {\mathbb {T}}^2\rightarrow {\mathbb {R}}^3$ is an $(A_0,V_0,c_0)$-Helfrich flow starting in $f_0$ with $f(t)=f_{\gamma (t)}$ for 0≦t<T and a smooth family of immersions $\gamma :[0,T)\times {\mathbb {S}}^1\rightarrow {\mathbb {H}}^2$, then

$$\begin{aligned} \sup _{0\leqq t<T} {\mathcal {L}}_{{\mathbb {H}}^2}(\gamma (t)) < \infty . \end{aligned}$$

### Proof

Firstly, proceeding as in (4.4) and (4.5), using Remark 2.4 and (2.4), one finds that

8.2 $$\begin{aligned} 0<\gamma ^{(2)}(t,\cdot )\leqq \frac{1}{2}\textrm{diam}(f(t)) \le C(A_0,c_0,{\mathcal {H}}_{c_0}(f_0)). \end{aligned}$$

Moreover, using [19, Lemma 2.5] with (2.4) and Remark 2.4, also

$$\begin{aligned} {\mathcal {L}}(\gamma (t)) = \int _{{\mathbb {S}}^1}|\partial _u \gamma (t,u)|\textrm{d}u \leqq C(A_0,c_0,{\mathcal {H}}_{c_0}(f_0)). \end{aligned}$$

For the sake of contradiction, suppose that $\limsup _{t\nearrow T}{\mathcal {L}}_{{\mathbb {H}}^2}(\gamma (t))=\infty$. Revisiting (8.1), the above yields $\liminf _{t\nearrow T} \min _{u\in {\mathbb {S}}^1}\gamma ^{(2)}(t,u) = 0$. So choose sequences $t_j\nearrow T$ and $u_j\in {\mathbb {S}}^1$ with $\gamma ^{(2)}(t_j,u_j)\rightarrow 0$ and write $z_j=f(t_j,(u_j,0))\in {\mathbb {R}}^3$. Next, denote by $V_j$ the oriented varifold in ${\mathbb {R}}^3$ induced by $f_{\gamma (t_j)}$, cf. [43, Section 2.3]. Using Proposition 2.9 and Remark 2.4, each $f_{\gamma (t_j)}$ is an embedding and therefore each $V_j$ belongs to the class of volume varifolds in [51, Definition 7.4]. By (2.2),

$$\begin{aligned} \int _{{\mathbb {T}}^2}|A|^2\textrm{d}\mu = 4 {\mathcal {W}}(f(t)) \leqq C(A_0,c_0,{\mathcal {H}}_{c_0}(f_0)), \end{aligned}$$

using also (2.4) and Remark 2.4. Due to (8.2), one can choose $h_j\in {\mathbb {R}}$ such that, for some compact $K\subseteq {\mathbb {R}}^3$, $\textrm{supp}(V_j-(h_j,0,0))\subseteq K$ for all $j\in {\mathbb {N}}$.

Thus, by [51, Theorem 7.6], after passing to a subsequence without relabeling, $(z_j-(h_j,0,0))\rightarrow z^*\in {\mathbb {R}}\times \{0\}^2$, $(V_j-(h_j,0,0))\rightarrow V$ as oriented varifolds for a volume varifold *V*, and

$$\begin{aligned} {\mathcal {H}}_{c_0}(V)\leqq \liminf _{j\rightarrow \infty }{\mathcal {H}}_{c_0}(V_j) < \Omega (A_0,V_0,c_0). \end{aligned}$$

Particularly, using [43, Corollary 4.11],

8.3 $$\begin{aligned} \theta ^2(\mu _V,z) < 2\quad \hbox { for all}\ z\in {\mathbb {R}}^3. \end{aligned}$$

Consider now reparametrizations ${\widetilde{\gamma }}_j:{\mathbb {S}}^1\rightarrow {\mathbb {H}}^2$ of $\gamma (t_j)-(h_j,0)$ by constant Euclidean speed, that is, such that $\partial _u|\partial _u{\widetilde{\gamma }}_j|=0$ on ${\mathbb {S}}^1$. After passing to a further subsequence without relabeling, using [52, Lemma 3.7],

$$\begin{aligned}&{\widetilde{\gamma }}_j \rightharpoonup ^* {\widetilde{\gamma }}\text { in }W^{1,\infty }({\mathbb {S}}^1) \text { and } {\widetilde{\gamma }}_j \rightarrow {\widetilde{\gamma }}\quad \text {uniformly};\\&{\widetilde{\gamma }}_j \rightharpoonup {\widetilde{\gamma }}\quad \text {in }W^{2,2}([a,b])\text { for every } [a,b]\subseteq \{{\widetilde{\gamma }}^{(2)}>0\} \end{aligned}$$

for some ${\widetilde{\gamma }}\in W^{1,\infty }({\mathbb {S}}^1,{\mathbb {R}}\times [0,\infty ))$. Now proceeding as in [52, proof of Proposition 4.2], one argues that, for $z^*=\lim _{j\rightarrow \infty }(z_j-(h_j,0,0))\in {\mathbb {R}}\times \{0\}^2$,

$$\begin{aligned} \theta ^2(\mu _V,z^*)\geqq 2, \end{aligned}$$

contradicting (8.3). □

### Proof of Theorem 1.2

As for Theorem 1.1, one may without loss of generality assume (5.7) for some $\eta \in (0,1)$. Then consider a concentration limit ${\hat{f}}:{\hat{\Sigma }}\rightarrow {\mathbb {R}}^3$ as in Proposition 5.6 and proceed as in [19, proof of Theorem 1.2] to deduce from Proposition 8.2 that ${\hat{\Sigma }}$ is necessarily compact and r>0. Clearly $H_{{\hat{f}}}\not \equiv const$ by Aleksandrov’s theorem [2]. So the claim follows from Lemma 6.3, arguing as in [19, Lemma 3.4]. □

## Data Availability

Data sharing not applicable to this article as no datasets were generated or analyzed during the current study.

## Appendix A. Proof of Lemma 3.1

This section is devoted to proving Lemma 3.1. We first compute the exact localized evolution of the energy.

### Lemma A.1

Let $A_0,V_0>0$ satisfy (1.9), $c_0\in {\mathbb {R}}$ and $f:[0,T)\times \Sigma \rightarrow {\mathbb {R}}^3$ be an $(A_0,V_0,c_0)$-Helfrich flow. If ${\widetilde{\eta }}\in C_c^{\infty }({\mathbb {R}}^3)$ and $\eta ={\widetilde{\eta }}\circ f$,

$$\begin{aligned} \frac{\textrm{d}}{\textrm{d}t}&\int _{\Sigma }\frac{1}{2} H^2\eta \textrm{d}\mu + \int _{\Sigma } |\nabla {\mathcal {W}}_0(f)|^2\eta \textrm{d}\mu \\&= (\lambda _1+\frac{1}{2}c_0^2)\int _{\Sigma }\Delta H H\eta \textrm{d}\mu + \int _{\Sigma }\big ((\lambda _1+\frac{1}{2}c_0^2)H+\lambda _2\big )|A^0|^2H\eta \textrm{d}\mu \\&\quad -2\int _{\Sigma }\langle \nabla {\mathcal {W}}_0(f),\nu \rangle \langle \nabla H,\nabla \eta \rangle _{g_f}\textrm{d}\mu - \int _{\Sigma }\langle \nabla {\mathcal {W}}_0(f),\nu \rangle H\Delta \eta \textrm{d}\mu \\&\quad + \frac{1}{2}\int _{\Sigma }H^2\partial _t\eta \textrm{d}\mu \\&\quad +c_0\int _{\Sigma }(|A^0|^2-\frac{1}{2}H^2)(\langle \nabla {\mathcal {W}}_0(f),\nu \rangle \eta + 2\langle \nabla H,\nabla \eta \rangle _{g_f}+H\Delta \eta )\textrm{d}\mu \end{aligned}$$

as well as

$$\begin{aligned} \partial _t&\int _{\Sigma }|A^0|^2\eta \textrm{d}\mu +\int _{\Sigma }|\nabla {\mathcal {W}}_0(f)|^2\eta \textrm{d}\mu \\&= (2\lambda _1+c_0^2)\int _{\Sigma }\langle \nabla ^2H,A^0\rangle _{g_f}\eta \textrm{d}\mu + \int _{\Sigma }\big ((\lambda _1+\frac{1}{2}c_0^2)H+\lambda _2\big )|A^0|^2H\eta \textrm{d}\mu \\&\quad -2\int _{\Sigma }\langle \nabla {\mathcal {W}}_0(f),\nu \rangle \bigl (\langle A^0,\nabla ^2\eta \rangle _{g_f}+\langle \nabla H,\nabla \eta \rangle _{g_f}\bigr )\textrm{d}\mu \\&\quad + c_0\int _{\Sigma }(|A^0|^2-\frac{1}{2}H^2) \bigl ( \langle \nabla {\mathcal {W}}_0(f),\nu \rangle \eta + 2\langle A^0,\nabla ^2\eta \rangle _{g_f}+2\langle \nabla H,\nabla \eta \rangle _{g_f} \bigr )\textrm{d}\mu \\&\quad + \int _{\Sigma }|A^0|^2\partial _t\eta \textrm{d}\mu . \end{aligned}$$

### Proof

Using (2.5), (2.6) and [29, (31) and (32)], writing $\partial _tf=\xi \nu$,

$$\begin{aligned} \partial _t&\int _{\Sigma }\frac{1}{2}H^2\eta \textrm{d}\mu + \int _{\Sigma }|\nabla {\mathcal {W}}_0(f)|^2\eta \textrm{d}\mu \\&= \int _{\Sigma }\big ((\lambda _1+\frac{1}{2}c_0^2)H+\lambda _2\big )(\Delta H+|A^0|^2H)\eta \textrm{d}\mu \\&\quad +\int _{\Sigma } (2\xi \langle \nabla H,\nabla \eta \rangle _{g_f}+H\xi \Delta \eta )\textrm{d}\mu \\&\quad +c_0\int _{\Sigma }(|A^0|^2-\frac{1}{2}H^2)(\Delta H+|A^0|^2H)\eta \textrm{d}\mu +\frac{1}{2}\int _{\Sigma }H^2\partial _t\eta \textrm{d}\mu , \end{aligned}$$

since $\xi =-(\Delta H+|A^0|^2\,H)+c_0(|A^0|^2-\frac{1}{2}H^2)+(\lambda _1+\frac{1}{2}c_0^2)H+\lambda _2$. Moreover, also using $\Delta (H\eta ) = \Delta H\eta + 2\langle \nabla H,\nabla \eta \rangle _{g_f}+H\Delta \eta$, one finds

$$\begin{aligned} \partial _t&\int _{\Sigma }\frac{1}{2}H^2\eta \textrm{d}\mu + \int _{\Sigma }|\nabla {\mathcal {W}}_0(f)|^2\eta \textrm{d}\mu \\&= \int _{\Sigma }\big ((\lambda _1+\frac{1}{2}c_0^2)H+\lambda _2\big )(\Delta (H\eta )+|A^0|^2H\eta )\textrm{d}\mu \\&\quad -2\int _{\Sigma }\langle \nabla {\mathcal {W}}_0(f),\nu \rangle \langle \nabla H,\nabla \eta \rangle _{g_f}\textrm{d}\mu -\int _{\Sigma }\langle \nabla {\mathcal {W}}_0(f),\nu \rangle H\Delta \eta \textrm{d}\mu \\&\quad +c_0\int _{\Sigma }(|A^0|^2-\frac{1}{2}H^2)(\langle \nabla {\mathcal {W}}_0(f),\nu \rangle \eta + 2\langle \nabla H,\nabla \eta \rangle _{g_f}+H\Delta \eta )\textrm{d}\mu \\&\quad +\frac{1}{2}\int _{\Sigma }H^2\partial _t\eta \textrm{d}\mu . \end{aligned}$$

The first claim follows after integrating by parts in the first term on the right. Moreover, as in [46, Article C, proof of Lemma 2.8] and [29, p. 423], one finds

$$\begin{aligned} \partial _t(|A^0|^2\textrm{d}\mu )&= 2\nabla _i(\nabla _j\xi A^0(e_i,e_j))\textrm{d}\mu -\nabla _j\xi \nabla _jH\textrm{d}\mu +|A^0|^2H\xi \textrm{d}\mu \\&=2\nabla _i(\nabla _j\xi A^0(e_i,e_j))\textrm{d}\mu -\nabla _j(\xi \nabla _jH)\textrm{d}\mu +(\Delta H+|A^0|^2H)\xi \textrm{d}\mu . \end{aligned}$$

Again, with $$\xi =-(\Delta H+|A^0|^2\,H)+c_0(|A^0|^2-\frac{1}{2}H^2)+(\lambda _1+\frac{1}{2}c_0^2)H+\lambda _2$$,

$$\begin{aligned} \partial _t&(|A^0|^2\textrm{d}\mu ) + |\nabla {\mathcal {W}}_0(f)|^2\textrm{d}\mu \\&= 2\nabla _i(\nabla _j\xi A^0(e_i,e_j))\textrm{d}\mu -\nabla _j(\xi \nabla _jH)\textrm{d}\mu \\&\quad + c_0(|A^0|^2-\frac{1}{2}H^2)\langle \nabla {\mathcal {W}}_0(f),\nu \rangle \textrm{d}\mu +\big ((\lambda _1+\frac{1}{2}c_0^2)H+\lambda _2\big )\langle \nabla {\mathcal {W}}_0(f),\nu \rangle \textrm{d}\mu . \end{aligned}$$

Integrating by parts and using $\nabla _iH=2(\nabla _jA^0)_{ij}$ by Codazzi–Mainardi, one obtains

$$\begin{aligned} \partial _t&\int _{\Sigma }|A^0|^2\eta \textrm{d}\mu +\int _{\Sigma }|\nabla {\mathcal {W}}_0(f)|^2\eta \textrm{d}\mu \\&= \int _{\Sigma } 2\xi A^0_{ij}\nabla _{ij}^2\eta +2\xi \nabla _jH\nabla _j\eta + \big ((\lambda _1+\frac{1}{2}c_0^2)H+\lambda _2\big )\langle \nabla {\mathcal {W}}_0(f),\nu \rangle \eta \textrm{d}\mu \\&\quad +\int _{\Sigma }c_0(|A^0|^2-\frac{1}{2}H^2)\langle \nabla {\mathcal {W}}_0(f),\nu \rangle \eta \textrm{d}\mu + \int _{\Sigma } |A^0|^2\partial _t\eta \textrm{d}\mu . \end{aligned}$$

Integration by parts yields

$$\begin{aligned}  &   \int _{\Sigma }\big ((\lambda _1+\frac{1}{2}c_0^2)H+\lambda _2\big )(2A^0_{ij}\nabla ^2_{ij}\eta +2\nabla _jH\nabla _j\eta +\Delta H\eta )\textrm{d}\mu \\  &   \quad = (2\lambda _1+c_0^2)\int _{\Sigma }\langle \nabla ^2H,A^0\rangle _{g_f}\eta \textrm{d}\mu , \end{aligned}$$

so that the claim follows. □

### Proof of Lemma 3.1

Using (2.2) and Lemma A.1 with ${\tilde{\eta }}:= {\tilde{\gamma }}^4$, so that $\eta = \gamma ^4$, one finds

$$\begin{aligned}&\frac{\textrm{d}}{\textrm{d}t}\int _{\Sigma }|A|^2\gamma ^4\textrm{d}\mu +2\int _{\Sigma }|\nabla {\mathcal {W}}_0(f)|^2\gamma ^4\textrm{d}\mu \\&\quad = (2\lambda _1+c_0^2)\int _{\Sigma }\bigl (\langle \nabla ^2H,A\rangle _{g_f}+|A^0|^2H^2\bigr )\gamma ^4\textrm{d}\mu +2\lambda _2 \int _{\Sigma }|A^0|^2H\gamma ^4\textrm{d}\mu \\&\quad \quad -2\int _{\Sigma }\langle \nabla {\mathcal {W}}_0(f),\nu \rangle \bigl (2\langle \nabla H,\nabla \gamma ^4\rangle _{g_f}+\langle \nabla ^2\gamma ^4,A\rangle _{g_f}\bigr )\textrm{d}\mu +\int _{\Sigma }|A|^2\partial _t\gamma ^4\textrm{d}\mu \\&\qquad + c_0\int _{\Sigma }(|A^0|^2\!-\!\frac{1}{2}H^2)\bigl ( 2\langle \nabla ^2\gamma ^4,A\rangle _{g_f}\!+\!4\langle \nabla H,\nabla \gamma ^4\rangle _{g_f}\\&\qquad +2\langle \nabla {\mathcal {W}}_0(f),\nu \rangle \gamma ^4 \bigr )\textrm{d}\mu . \end{aligned}$$

We now estimate the individual terms starting with $\int _\Sigma |A|^2\partial _t\gamma ^4\textrm{d}\mu$. A straight-forward computation yields

$$\begin{aligned} |\partial _t\gamma ^4|\leqq C\Lambda \gamma ^3\bigl ( |\nabla {\mathcal {W}}_0(f)|+(|\lambda _1|+c_0^2)|A|+|\lambda _2|+|c_0||A|^2 \bigr ). \end{aligned}$$

The last term arising in $\int _\Sigma |A|^2 \partial _t \gamma ^4 \textrm{d}\mu$ is estimated by

$$\begin{aligned} C\Lambda |c_0|\int _\Sigma |A|^4\gamma ^3\textrm{d}\mu \leqq C c_0^2\int _\Sigma |A|^4\gamma ^4\textrm{d}\mu + C\Lambda ^2\int _\Sigma |A|^4\gamma ^2\textrm{d}\mu \end{aligned}$$

whereas the first term can be absorbed via

$$\begin{aligned} C\Lambda \int _\Sigma |A|^2|\nabla {\mathcal {W}}_0(f)|\gamma ^3\textrm{d}\mu \le \varepsilon \int _\Sigma |\nabla {\mathcal {W}}_0(f)|^2\gamma ^4\textrm{d}\mu +C(\varepsilon )\Lambda ^2\int _{\Sigma }|A|^4\gamma ^2\textrm{d}\mu . \end{aligned}$$

Moreover, as $$|\nabla ^2\gamma ^4|\le C(\Lambda ^2+|A|\Lambda \gamma )\gamma ^2$$,

$$\begin{aligned} \int _{\Sigma } |\nabla {\mathcal {W}}_0(f)||\nabla ^2\gamma ^4||A|\textrm{d}\mu&\le \varepsilon \int _{\Sigma }|\nabla {\mathcal {W}}_0(f)|^2\gamma ^4\textrm{d}\mu + C(\varepsilon )\Lambda ^2\int _{\Sigma }|A|^4\gamma ^2\textrm{d}\mu \\&\quad +C(\varepsilon )\Lambda ^4\int _{\{\gamma >0\}}|A|^2\textrm{d}\mu . \end{aligned}$$

Furthermore, as in [29, proof of Lemma 3.2], one obtains

A.1 $$\begin{aligned} \int _{\Sigma }|\nabla H|^2\gamma ^2\textrm{d}\mu&\leqq \varepsilon \int _{\Sigma } |\nabla {\mathcal {W}}_0(f)|^2\gamma ^4\textrm{d}\mu \nonumber \\&\quad + C\Big (\Lambda ^2+\frac{1}{\varepsilon }\Big )\int _{\{\gamma >0\}} H^2\textrm{d}\mu + C\int _{\Sigma }|A|^4\gamma ^2\textrm{d}\mu . \end{aligned}$$

Therefore, using $$|\nabla \gamma ^4|\leqq C\gamma ^3\Lambda $$,

$$\begin{aligned} \int _{\Sigma }|\nabla {\mathcal {W}}_0(f)||\nabla H||\nabla \gamma ^4|\textrm{d}\mu&\leqq \varepsilon \int _{\Sigma }|\nabla {\mathcal {W}}_0(f)|^2\gamma ^4\textrm{d}\mu +C(\varepsilon )\Lambda ^2\int _{\Sigma }|A|^4\gamma ^2\textrm{d}\mu \\&\quad +C(\varepsilon )\Lambda ^4\int _{\{\gamma >0\}}H^2\textrm{d}\mu . \end{aligned}$$

Using $|c_0(|A^0|^2-\frac{1}{2}H^2)|\leqq C |c_0||A|^2$ as well as $|\nabla ^2\gamma ^4|\leqq C(\Lambda ^2+|A|\Lambda \gamma )\gamma ^{2}$, the terms containing $c_0$ can be dealt with as follows. Firstly, we have

$$\begin{aligned} |c_0|\int _\Sigma |A|^3|\nabla ^2\gamma ^4|\textrm{d}\mu&\leqq C|c_0|\Lambda ^2\int _\Sigma |A|^3\gamma ^2\textrm{d}\mu + C|c_0|\Lambda \int _\Sigma |A|^4\gamma ^3\textrm{d}\mu \\&\leqq C c_0^2\int _\Sigma |A|^4\gamma ^4\textrm{d}\mu + C\Lambda ^4\int _{\{\gamma >0\}}|A|^2\textrm{d}\mu \\&\quad + C\Lambda ^2\int _\Sigma |A|^4\gamma ^2\textrm{d}\mu . \end{aligned}$$

Secondly, using (A.1),

$$\begin{aligned} |c_0|\int _\Sigma |A|^2|\nabla H||\nabla \gamma ^4|\textrm{d}\mu&\leqq Cc_0^2\int _\Sigma |A|^4\gamma ^4\textrm{d}\mu +\varepsilon \int _\Sigma |\nabla {\mathcal {W}}_0(f)|^2\gamma ^4 \textrm{d}\mu \\&\quad + C(\varepsilon )\Lambda ^2\int _\Sigma |A|^4\gamma ^2\textrm{d}\mu + C(\varepsilon )\Lambda ^4\int _{\{\gamma >0\}}|A|^2\textrm{d}\mu \end{aligned}$$

and finally,

$$\begin{aligned} |c_0|\int _\Sigma |A|^2|\nabla {\mathcal {W}}_0(f)|\gamma ^4\textrm{d}\mu \le \varepsilon \int _\Sigma |\nabla {\mathcal {W}}_0(f)|^2\gamma ^4\textrm{d}\mu + C(\varepsilon )c_0^2\int _\Sigma |A|^4\gamma ^4\textrm{d}\mu \end{aligned}$$

which completes the proof after taking ε>0 sufficiently small and absorbing. □

## Appendix B. On Preservation of Axisymmetry

Following [19, Definition 1.1] with the identification ${\mathbb {S}}^1={\mathbb {R}}\ /2\pi {\mathbb {Z}}$, a smooth immersion $f:{\mathbb {T}}^2\rightarrow {\mathbb {R}}^3$ is said to be an axisymmetric torus if, for a smooth immersion $\gamma :{\mathbb {S}}^1\rightarrow {\mathbb {H}}^2$,

$$\begin{aligned} f(u,v)=(\gamma ^{(1)}(u),\gamma ^{(2)}(u)\cos (v),\gamma ^{(2)}(u)\sin (v)) \end{aligned}$$

where ${\mathbb {T}}^2={\mathbb {S}}^1\times {\mathbb {S}}^1$. One has the following characterization:

### Proposition B.1

([19, Proposition 3.2]) A smooth immersion $f:{\mathbb {T}}^2\rightarrow {\mathbb {R}}^3$ is an axisymmetric torus if and only if

- $f(u,v+\varphi ) = R_{\varphi }f(u,v)$ for all $u,v,\varphi \in {\mathbb {S}}^1$ where
  $$\begin{aligned} R_{\varphi } = \begin{pmatrix} 1& 0& 0\\ 0& \cos \varphi & -\sin \varphi \\ 0& \sin \varphi & \cos \varphi \end{pmatrix}\quad \text {and} \end{aligned}$$
- $f^{(3)}(u,0)=0$ for all $u\in {\mathbb {S}}^1$ and $f^{(2)}(u_0,0)\geqq 0$ for at least one $u_0\in {\mathbb {S}}^1$.

For Lemma 8.1, if one followed the proof of [19, Lemma 3.3] line-by-line, in the case of the Helfrich flow, one would have to use Corollary 5.4 and Proposition 5.2 in place of [30, Theorem 1.2 and Equation (4.27)]. Then however, one needs to additionally assume (1.10). Therefore, we repeat the general strategy of the proof of [19, Lemma 3.3] but avoid using Corollary 5.4 and Proposition 5.2, that is, do not assume (1.10).

### Proof of Lemma 8.1

Let $f:[0,T)\times {\mathbb {T}}^2\rightarrow {\mathbb {R}}^3$ be an $(A_0,V_0,c_0)$-Helfrich flow starting in an axisymmetric torus $f_0=f_{\gamma _0}$ as in the statement. We first verify Proposition B.1(a) along the evolution. To this end, for fixed $\varphi \in {\mathbb {S}}^1$, $R_{\varphi }:{\mathbb {R}}^3\rightarrow {\mathbb {R}}^3$ is an isometry. Therefore, one readily checks that $(R_{\varphi }^{-1})f(t,\cdot +(0,\varphi )):[0,T)\times {\mathbb {T}}^2\rightarrow {\mathbb {R}}^3$ is an $(A_0,V_0,c_0)$-Helfrich flow starting in $R_{\varphi }^{-1}f_0(\cdot +(0,\varphi ))=f_0$, using that $f_0$ is an axisymmetric torus. Well-posedness of (2.7), see [28], then yields that each *f*(*t*) satisfies Proposition B.1(a) for 0≦t<T.

Therefore, there exist smooth functions $x,y,z:[0,T)\times {\mathbb {S}}^1\rightarrow {\mathbb {R}}$ with

B.1 $$\begin{aligned} f(t,(u,v)) = R_vf(t,(u,0)) = R_v \big (x(t,u),y(t,u),z(t,u)\big ). \end{aligned}$$

In order to establish Proposition B.1(b), we first show z≡0 by slightly modifying the proof of [19, Lemma 3.3]. Consider the normal field

B.2 $$\begin{aligned} {\widetilde{\nu }} := \frac{\partial _uf\times \partial _vf}{|\partial _uf\times \partial _vf|} = \frac{1}{\sqrt{\det (Df^tDf)}} R_v\begin{pmatrix} y\partial _uy+z\partial _uz\\ -y\partial _ux\\ -z\partial _ux \end{pmatrix}. \end{aligned}$$

For the sake of contradiction, suppose that

B.3 $$\begin{aligned} S=\sup \{s\in [0,T):f(t)\text { is an axisymmetric torus for all }0\leqq t\leqq s\}<T. \end{aligned}$$

As in the proof of [19, Lemma 3.3], *f*(*S*) is an axisymmetric torus and z(S,·)=0. Moreover, since $\partial _tf = \langle \partial _tf,\nu \rangle \nu$, (B.1) and (B.2) yield that on $[0,T)\times {\mathbb {S}}^1$ we have

$$\begin{aligned} \partial _t z^2 = 2z\partial _tz=\left. 2z\langle \partial _t f,{\widetilde{\nu }}\rangle {\widetilde{\nu }}^{(3)}\right| _{v=0} = \left. 2z \langle \partial _tf,{\widetilde{\nu }}\rangle \frac{1}{\sqrt{\det (Df^tDf)}}\right| _{v=0} (-z\partial _ux). \end{aligned}$$

For 0<ε<T-S, since *f* is a *smooth* family of *immersions* on $[S,S+\varepsilon ]\times {\mathbb {T}}^2$, we find

$$\begin{aligned}&\partial _t z^2 \leqq \sup _{t\in [S,S+\varepsilon ],u\in {\mathbb {S}}^1} \Big |\frac{2\langle \partial _tf(t,(u,0)),{\widetilde{\nu }}(t,(u,0))\rangle \partial _ux(t,u)}{\sqrt{\det [Df^t(t,(u,0))Df(t,(u,0))]}}\Big | \cdot z^2=:C\cdot z^2\quad \\&\quad \hbox { on}\ [S,S+\varepsilon ]\times {\mathbb {S}}^1. \end{aligned}$$

Since $z(S,\cdot )\equiv 0$, this yields z(t,u)=0 for all $S\leqq t\leqq S+\varepsilon$ and $u\in {\mathbb {S}}^1$.

Notice that y(S,·)>0 since *f*(*S*) is an axisymmetric torus. Therefore, for ε>0 small enough, there exists $u_0\in {\mathbb {S}}^1$ such that $y(t,u_0)>0$ for all $S\leqq t \leqq S + \varepsilon$. Thus, by Proposition B.1, *f*(*t*) is an axisymmetric torus for $t\in [0,S+\varepsilon ]$, contradicting (B.3). □
